# Implementing Maternal Death Surveillance and Response in Kenya: Incremental Progress and Lessons Learned

**DOI:** 10.9745/GHSP-D-17-00130

**Published:** 2017-09-27

**Authors:** Helen Smith, Charles Ameh, Pamela Godia, Judith Maua, Kigen Bartilol, Patrick Amoth, Matthews Mathai, Nynke van den Broek

**Affiliations:** a Centre for Maternal and Newborn Health, Liverpool School of Tropical Medicine, Liverpool, UK.; b Liverpool School of Tropical Medicine Kenya Office, Nairobi, Kenya.; c Kenya National Maternal and Perinatal Death Surveillance and Response Secretariat, Nairobi, Kenya.; d Reproductive Maternal Health Services Unit, Ministry of Health, Nairobi, Kenya.; e Division of Family Health, Ministry of Health, Nairobi, Kenya.

## Abstract

Maternal death surveillance and response (MDSR) constitutes a quality improvement approach to identify how many maternal deaths occur, what the underlying causes of death and associated factors are, and how to implement actions to reduce the number of preventable stillbirths and maternal and neonatal deaths. This requires a coordinated approach, ensuring both national- and district-level stakeholders are enabled and supported and can implement MDSR in a “no name, no blame” environment. This field action report from Kenya provides an example of how MDSR can be implemented in a “real-life” setting by summarizing the experiences and challenges faced thus far by maternal death assessors and Ministry of Health representatives in implementing MDSR. Strong national leadership via a coordinating secretariat has worked well in Kenya. However, several challenges were encountered including underreporting of data, difficulties with reviewing the data, and suboptimal aggregation of data on cause of death. To ensure progress toward a full national enquiry of all maternal deaths, we recommend improving the notification of maternal deaths, ensuring regular audits and feedback at referral hospitals lead to continuous quality improvement, and strengthening community linkages with health facilities to expedite maternal death reporting. Ultimately, both a top-down and bottom-up approach is needed to ensure success of an MDSR system. Perinatal death surveillance and response is planned as a next phase of MDSR implementation in Kenya. To ensure the process continues to evolve into a full national enquiry of all maternal deaths, we recommend securing longer-term budget allocation and financial commitment from the ministry, securing a national legal framework for MDSR, and improving processes at the subnational level.

## Abstract (precis)

A national coordinating structure was established but encountered significant challenges including: (1) a low number of estimated maternal deaths identified that only included some occurring within facilities, (2) only half of those identified were reviewed, (3) reviewers had difficulties assessing the cause of death largely because of limited documentation in clinical records; and (4) resulting actions were limited. Successful implementation will require addressing many issues, including building support for the process lower down in the health system.

## BACKGROUND

In 2012, the World Health Organization (WHO) and partners introduced the Maternal Death Surveillance and Response (MDSR) approach as a new method to maternal death review.^1^ While MDSR was built upon well-established review processes, its benefit was that it reemphasized the importance of the timely reporting (surveillance) of deaths and implementation of actions (response) to prevent further deaths. Maternal mortality is often described as the “litmus test” of the health system – a measure of a system's ability to respond to women's health needs, especially during and after pregnancy and birth.^2^ In principle, MDSR builds on existing health system processes for reporting and surveillance, and offers a systematic way of ensuring information on avoidable factors is aggregated and used to guide action at all levels.^1^ Implementing MDSR involves establishing an entire system to link surveillance and review of maternal deaths at facility and community levels in order to inform national scale in-depth confidential enquiry of maternal deaths. Depending on the burden of maternal mortality, some countries may prefer to also include surveillance and review of near-miss and perinatal deaths at national or subnational levels. Because existing approaches, systems, or platforms for capturing maternal health data are similarly named – Maternal Death Review (MDR), MDSR, and Maternal and Perinatal Death Surveillance and Response (MPDSR) – they are sometimes used interchangeably, which can lead to confusion.

Maternal mortality is often described as the “litmus” test of the health system.

The first WHO report on the global implementation of MDSR clearly set out the key policy indicators and principles to guide operation of the system (Box 1). The indicators were measured in the 2015 MDSR baseline survey, which found that of 67 countries surveyed 86% had a national policy to notify all maternal deaths, 85% had a national policy to review all maternal deaths, 76% had a national maternal death review committee in place, 65% had subnational maternal death review committees in place, and 60% had both national and subnational committees, but only 46% had national maternal death review committees that met at least biannually.^3^ While this represents good progress over a relatively short period, these figures mask the wide variation across countries in the adoption of each of the key components of the MDSR. The challenge of agreeing upon national policy indicators for MDSR is that it does not ensure the adoption of processes and principles to guide operation at subnational level.

BOX 1 **Key Components of a National Maternal Death Surveillance and Response (MDSR) System**

**Key policy indicators:**

- A national policy to notify all maternal deaths
- A national policy to review all maternal deaths
- A national maternal death review committee in place
- Subnational maternal death review committees in place
- Both national and subnational maternal death review committees in place
- A national maternal death review committee that meets at least biannually

**Key principles to guide operation of the system:**

- Notification and investigation of all suspected maternal deaths in women of reproductive age (15–45 years)
- Notification within 24 hours of maternal deaths in facilities (or within 48 hours when a woman dies in the community)
- Zero reporting when no suspected maternal deaths have occurred
- Timely review of all probable maternal deaths
- Immediate recommendations, where possible, to help facilities and communities prevent similar deaths, ensuring that key messages reach people who can make a difference
- Timely review and analysis at district and national levels to identify trends and patterns
- Timely publication of findings and recommendations at national level
- Continuous monitoring of the MDSR system and of how recommendations are implemented

Source: World Health Organization, 2016.^3^

MDSR requires identification and reporting pathways for maternal deaths, review of deaths, aggregation of data, interpretation of findings, and formulation and implementation of recommendations for action and quality improvement at each level of the health system. To be effective, MDSR needs central and local government support, adequate human and financial resources, and stakeholder participation and buy-in, including a “no name, no blame” approach to maternal death review that emphasizes identifying and correcting health system problems rather than faults in individuals' practice and management. A legal framework is also essential to ensure that maternal death reporting is mandatory and, perhaps more importantly, that information generated as part of the MDSR is not used for litigation purposes, for which separate processes exist. Depending on the country context, some or all of these factors may be missing or require strengthening. In practice, countries tend to start by introducing components of MDSR at different times, making full implementation incremental rather than rapid and linear. For these reasons, it has been recommended that countries start by implementing elements of the MDSR system in specific projects, and scaling up and introducing perinatal deaths only when systems and processes are in place and when success is demonstrated.^3^

Maternal death surveillance and response (MDSR) requires identification and reporting pathways for maternal deaths, review of deaths, aggregation of data, interpretation of findings, and formulation and implementation of recommendations for action.

In this field action report, we describe the implementation of MDSR in Kenya, which started with facility-based maternal death reviews and has progressed to a centrally coordinated system to support the development and establishment of a full confidential enquiry into maternal deaths process. We summarize the experiences and critical challenges faced thus far, and suggest improvements to overcome them. We also summarize program experiences and lessons learned though discussion with maternal death assessors and Ministry of Health (MOH) representatives during training workshops held in Kenya in June 2016. Assessors and MOH representatives were asked to reflect on the introduction of MDSR in Kenya and their experience of key steps in the process: establishing a national secretariat, retrieving case notes, and a confidential review of maternal deaths and assessors' personal experiences. For each step, stakeholders were asked to discuss in small groups the challenges faced, lessons learned, and suggestions for sustainability. We have drawn on these insights to highlight the challenges, lessons learned, and the way forward, which will be useful to other countries considering or setting up such a process.

## MATERNAL DEATH REVIEWS IN KENYA: AN OVERVIEW OF PROGRESS

In response to the high maternal mortality ratio, estimated to be 759 per 100,000 live births in 2000,^4^ the Government of Kenya made maternal death notification mandatory and introduced MDR in 2004. A review of all maternal deaths notified and reviewed between 2004 and 2006 revealed significant underreporting—only 46% of deaths reported via the Health Management Information System (HMIS) were notified via the MDR system.^5^ As a result, in 2009, the Government relaunched facility-based MDR, emphasizing a “no name, no blame” approach in order to increase facility and practitioner engagement. At this time, new supporting documents were also developed, including a revised notification form and a more detailed review form.^6^

In 2009, Kenya relaunched facility-based maternal death reviews, emphasizing a “no name, no blame” approach.

In 2011, a subsequent review of maternal deaths that occurred between 2008 and 2010 revealed a low review rate of just 20% of deaths recorded via the HMIS, poor completion of death review forms, lack of use of data to formulate recommendations, and no evidence of response to the findings of MDRs at facility or national levels. In response to this, and following the recommendations of the Commission on Information and Accountability in maternal and child health^7^ and the 2013 WHO technical guidelines for MDSR, the MOH re-orientated health care workers at all levels via workshops on MDSR.^8^ In 2014, a review of the MDR system showed mixed results; the system appeared to be working under partner-funded programs in a few counties, but notification and reporting of maternal deaths had not really improved overall.^9^ For example, the estimated number of maternal deaths in Kenya was 8,000, based on a maternal mortality rate (MMR) of 510 per 100,000 live births,^10^ compared with a total of 945 maternal deaths in facilities reported in the District Health Information System (DHIS) for the year 2014. Despite the MDSR tools—notification and review forms—being integrated into the DHIS database, the system has gaps, and the DHIS and Civil Registration and Vital Statistics system do not yet adequately capture all maternal deaths in Kenya.

National guidelines for Maternal and Perinatal Death Surveillance and Response (MPDSR) were launched in Kenya in 2016 (see Supplement 1).^11^ The guidelines were formulated to include perinatal death surveillance and response—hence the inclusion of the ‘P’ in MPDSR. The guidelines provide a framework for establishing and maintaining a system for collecting, analyzing, and reviewing data on stillbirths, neonatal and maternal deaths, and maternal near-misses.

**FIGURE 1:**
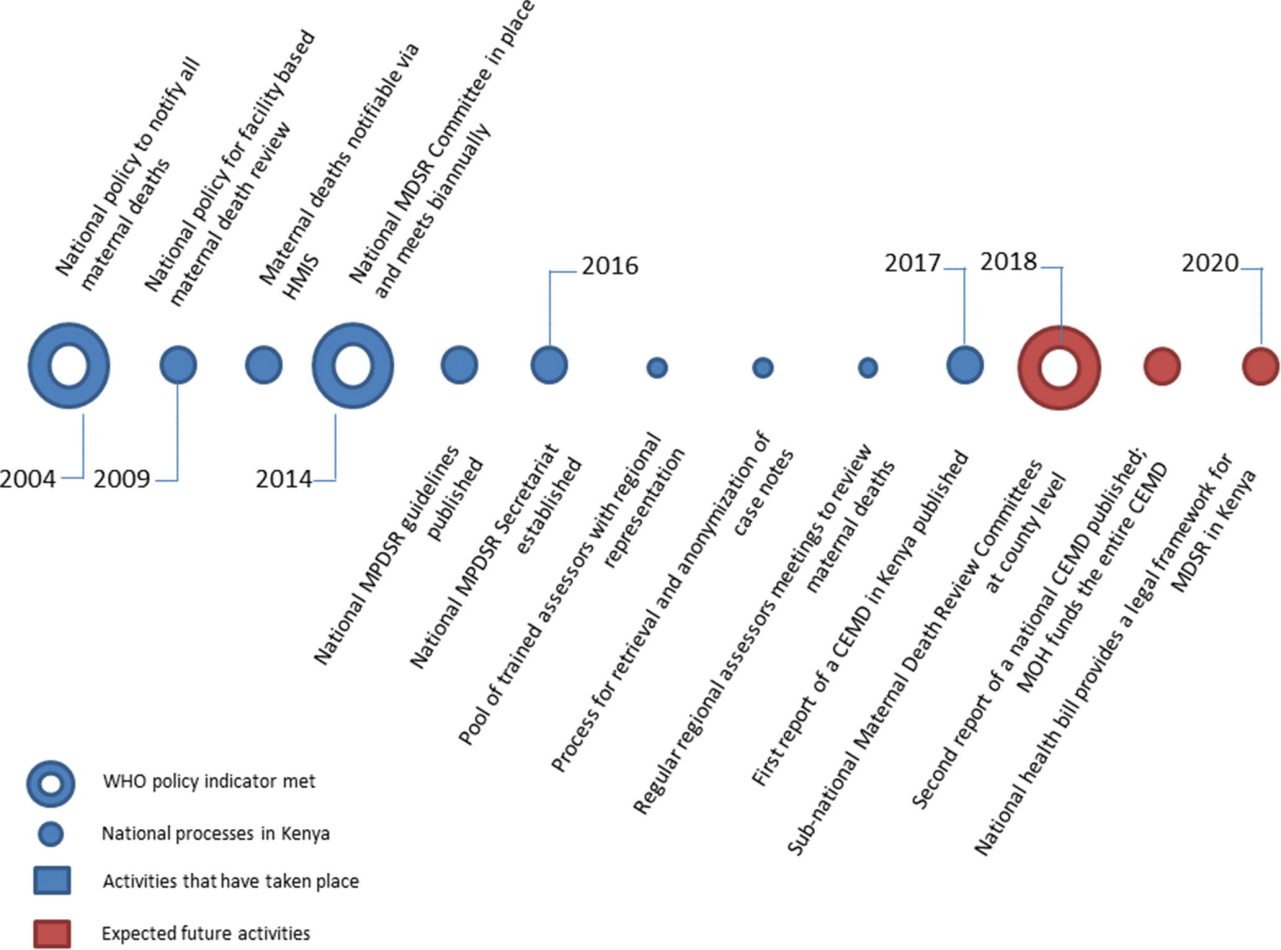
Timeline of Maternal Death Surveillance and Response Policies and Processes in Kenya Abbreviations: CEMD, Confidential Enquiry into Maternal Deaths; HMIS, Health Management Information System; MDSR, Maternal Death Surveillance and Response; MPDSR, Maternal and Perinatal Death Surveillance and Response; WHO, World Health Organization.

The timeline of key MDSR policies set out by WHO (Box 1) and implementation of processes in Kenya is illustrated in Figure 1. Two of the global policy indicators have been met—a national policy to notify all maternal deaths, in 2004, and a national committee for MPDSR, in 2014. The committee was inaugurated by the Cabinet Secretary for Health after 18 months of stakeholder consultations. The 10-year hiatus between the first national policy on notification and coordinated action on maternal death review is partly explained by a lack of funding, illustrating that sustained support is needed to introduce and establish a new surveillance system. A third MPDSR policy, subnational maternal death review committees at the county level, should be established by 2018.

In 2014, Kenya established a national committee for maternal and perinatal death surveillance and response (MPDSR).

## IMPLEMENTING MPDSR IN KENYA: LESSONS LEARNED

### National Leadership

Since 2007, the Centre for Maternal and Newborn Health at the Liverpool School of Tropical Medicine—working with the Reproductive Maternal Health Services Unit in the MOH and with funding from Department for International Development (DFID)/UKAid and the United Nations Population Fund (UNFPA)—has supported the Kenya MOH to improve implementation of facility-based MDR and, since 2014, to strengthen central coordination of MDSR. While the funding and technical support has been important, the crucial impetus and commitment that drives the process has come from the Cabinet Secretary for Health; professional medical, nursing, and midwifery associations; and medical and nursing regulatory bodies. These key stakeholders participate actively in the process and are represented on the national MPDSR committee, along with representatives from UNFPA, WHO, the United Nations Children's Fund (UNICEF), the U.S. Agency for International Development (USAID), DFID, and civil society groups. As in other countries, such as India, South Africa, and the UK, –the support of professional associations has been key.^3^^,^^12^^–^^14^

The composition of a national MPDSR committee is an important consideration, to ensure representation of key organizations able to capitalize on the political commitment and to oversee the practical work of establishing the national process for MPDSR. The MOH representatives and assessors we consulted stated that getting all partners to agree on creating a national-level committee and identifying potential members from government and private stakeholders was difficult, particularly because there was no precedent or experience of running such a committee. Accordingly, finding people with the know-how of running the committee at the national level was a challenge.

### The Role of the National MPDSR Secretariat

In 2014, a new national MPDSR secretariat was established within the MOH. The secretariat received short-term technical support from the UK and South Africa. A terms of reference for the secretariat was developed and agreed upon (Supplement 2) by the MOH and technical partners. Secretariat staff help to retrieve paper-based case notes from county- and subcounty-level facilities of women who died a maternal death. This is done by secretariat staff visiting participating health facilities and carefully recording the number of case notes retrieved against a list of the number of maternal deaths reported to have occurred at the facility. Photocopies of original case notes are made and sent to the secretariat office, with the original notes remaining on site.

The assessors and MOH representatives we consulted shared several critical challenges in retrieving case notes. Staff at the health facility are often reluctant to release the case files of women who have died and case notes may be missing and/or contain insufficient information. The stakeholders explained that these problems may arise because of a lack of awareness of the MPDSR process, lack of trust and suspicion about what the case notes will be used for, and, linked to this, the fear of blame and follow-up action against individuals because of the information contained within the reports. Others pointed out that there is no legal mandate in Kenya to retrieve case notes, so the process is entirely based on goodwill.

Facility staff are often reluctant to release case files of women who have died and case notes may be missing and/or incomplete.

Case notes are centrally collated and anonymized, and subsequently sent to assessors for review. Anonymization of retrieved case notes happens at the secretariat and is carried out, by hand, by staff specifically employed for the task. Our stakeholders described this as a time-consuming and meticulous process that requires trained and dedicated staff. The assessors described inefficiencies in the process, particularly in terms of the time taken to review case notes, as some are as long as 50 pages and many contain too much or too little anonymization.

### Assessor Experiences

To facilitate the first national confidential enquiry into maternal deaths process, a total of 93 assessors from across Kenya were identified and trained to carry out in-depth confidential review of maternal deaths. The assessors are self-motivated health care professionals—medical officers, obstetricians, pediatricians, midwives, anesthetists, and public health specialists—serving on a voluntary basis. They represent various professional bodies, including the Kenya Medical and Dentists Practitioners Board, Kenya Obstetrics and Gynecological Society, Nursing Council of Kenya, Kenya Nurses and Midwives Association, and Kenya Clinical Officers Association, as well as national teaching and referral hospitals. The assessors conduct independent detailed assessments of maternal deaths, assign cause of death using the WHO International Statistical Classification of Diseases for Maternal Mortality (ICD-MM) cause classification system,^15^ and are guided by technical experts from South Africa and the UK.^16^^,^^17^

The ICD-MM classification system is the standard tool to guide the collection, coding, tabulation, and reporting of maternal mortality. Maternal deaths are characterized and defined as due to direct or indirect causes; deaths during pregnancy, childbirth, and puerperium; or late maternal deaths. Assessors were trained by the MPDSR secretariat and the team from the Liverpool School of Tropical Medicine on how to complete the assessor maternal death forms and how to group the deaths, based on the ICD-MM system. They described the confidential review process as having a steep learning curve, and mentioned the time taken to participate in review meetings and the workload involved as too much and too detailed. Assessors also described the process of reviewing in detail the case notes of women who have died as an emotional experience; some assessors felt they needed to suppress their feelings about the deaths in order to remain professional. The assessors also expressed frustration at having to work with incomplete notes that contained scanty details of case management, and that antenatal and referral notes were often missing. They commented that these problems were because MDR was not yet institutionalized as an activity at the county or facility level and that doing so will require further training in the process of maternal death review and how it is linked to national MDSR.

### Identification and Review of Maternal Deaths at County Level

Alongside the national confidential enquiry process, efforts have been made to strengthen maternal death reporting and data-capture processes at county and subcounty levels. MPDSR data collection forms have been integrated into the second version of the DHIS (DHIS2) database, enabling the routine notification, uploading, collection, and analysis of maternal deaths data. However, in 2014, only about 12% of the estimated annual number of maternal deaths were notified through the DHIS2 system.^18^ The DHIS records only facility-based maternal deaths, which means deaths that occur in the community are not currently captured, nor is the process of verbal autopsy well-developed. Critical challenges to maternal death reporting and review include lack of understanding of how to operationalize the MPDSR guidelines at county and subcounty level and lack of MPDSR committees at county level and quality improvement committees at health facility level to conduct reviews. While there is some evidence of improved reporting of the number of maternal deaths that occur at health facility level for those deaths that have been reviewed, almost none uploaded completed MDR forms into the DHIS.

In 2014, only about 12% of the estimated annual number of maternal deaths were notified through the DHIS2.

## RECOMMENDATIONS FOR ACTION

The first comprehensive report of a review of maternal deaths in Kenya is due to be published in 2017, and will provide an in-depth analysis of the underlying and contributory causes of death, key findings relating to the quality of care provided to women who died, and recommendations for improving the quality of care for each major cause of death. In short, the report states that 484 (51.2%) of the 945 maternal deaths reported in the DHIS in 2014 were assessed; the sample included only maternal deaths that occurred in major referral public and private health facilities in all regions of Kenya during 2014. Of the 484 maternal deaths assessed, 447 (92.4%) received suboptimal care; 394 (81.4%) received sub-optimal care where different management could have made a difference to the outcome; and in 37 (7.6%) of the maternal deaths, the assessors could not identify any suboptimal care. The most frequent gaps in care of women who died at all levels of care were incorrect management when a correct diagnosis was made, infrequent monitoring, and prolonged abnormal observation noted but no action taken. Poor recordkeeping and documentation were noted in most cases of maternal death assessed. These findings, together with detailed analysis of the underlying and contributory causes of death, form the basis of recommendations for action at community, facility, district, and national levels.

Only about half of the maternal deaths reported in 2014 were assessed in the first report of a review of maternal deaths in Kenya.

Key cross-cutting recommendations include:

- Improving the quality of documentation of care provided to women
- Ensuring maternity care providers receive regular mandatory updates in emergency obstetric care, including adequate training at lower levels of care to improve capacity to resuscitate women and adhere to protocols for transfer of critically ill women
- Reviewing policies to ensure that maternity services are staffed by competent and experienced care providers 24 hours a day and 7 days a week

Specific policy-level recommendations for MDSR include:

- Improving the notification of maternal deaths
- Ensuring regular audits and feedback opportunities at referral hospitals lead to continuous quality improvement
- Strengthening community linkages with health facilities to expedite reporting of maternal deaths

The community-targeted recommendation refers to establishing and following the process for maternal death notification and review at the community level, as set out in the MPDSR guidelines. Community Health Volunteers (CHVs) and Community Health Extension Workers (CHEWs) are responsible for this process, which includes immediate notification of a death by the CHV, confirmation it is a maternal death by the CHEW, filing a death notification form by the link facility in charge, and uploading the information into the DHIS by the facility records officer. The entire process should be completed within 24 hours. The country now has, for the first time, evidence-based recommendations that will form the basis of a national response—the missing ‘R’ in MDSR in many countries. For each recommendation, the report lists the stakeholder group responsible for implementation along with a timeline, indicators for monitoring progress, and targets to be achieved.

## THE WAY FORWARD: TOP-DOWN AND BOTTOM-UP OPERATIONALIZATION OF MPDSR

In Kenya, the success of setting up the national coordination system for confidential review of identified maternal deaths has to some extent overshadowed the development of a bottom-up approach to ensure increased coverage. Yet the development and sustainability of a national confidential enquiry into maternal deaths process will ultimately rest upon both a top-down and bottom-up approach. This coordinated approach will need to ensure the identification of all maternal deaths at both facility and community levels; notification of those deaths by appropriate MPDSR committees at subcounty and county levels; routine facility- and community-based review of maternal deaths; timely uploading of review data into DHIS2 for analysis with aggregation of data; and central review and formulation of recommendations by national assessors and the secretariat.

Development and sustainability of a national confidential enquiry into maternal deaths process will ultimately rest upon both a top-down and bottom-up approach.

**FIGURE 2:**
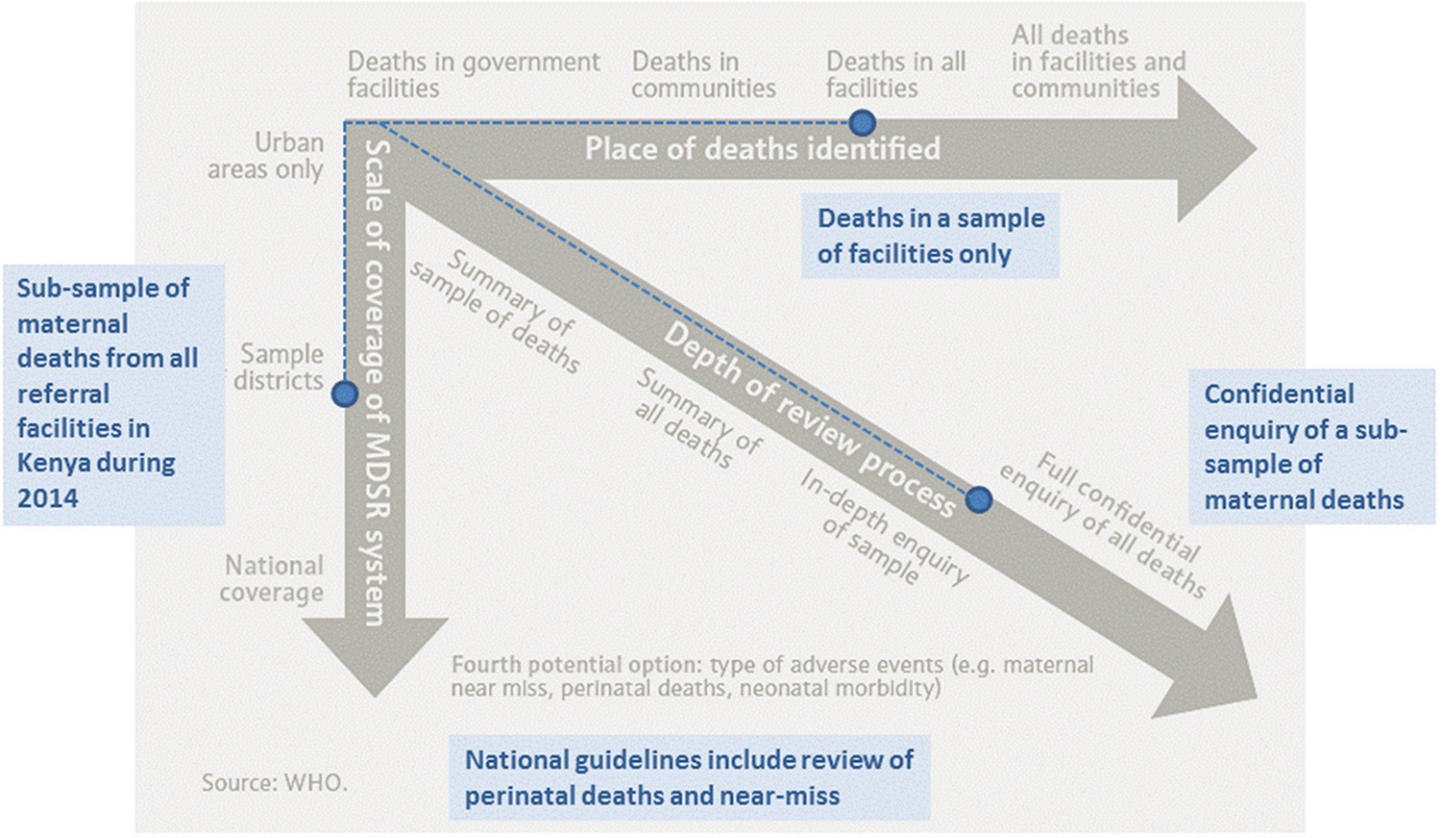
Implementation of Maternal and Perinatal Death Surveillance and Response in Kenya Mapped Onto the World Health Organization Phased Approach Abbreviation: MDSR, Maternal Death Surveillance and Response. The WHO phased approach^1^ is shown in gray, with implementation in Kenya shown in blue.

Figure 2 maps progress with MPDSR implementation in Kenya onto the phased approach recommended by WHO. It illustrates progress along all trajectories, with the potential to expand activities to include deaths in communities and full national coverage of surveillance and response over time. In Kenya, comparatively more progress has been achieved with regard to the depth of the review process, which was in some ways opportunistic, capitalizing on the availability of funding and technical support to establish the national MPDSR committee and secretariat. Since these national-level structures have been set up, rapid overall progress has taken place. However, there is still more to do. Progress in ensuring maternal death notification and review occurs at county level has been slower and more piecemeal. In addition, while the national MPDSR guidelines set out processes for surveillance and review of perinatal deaths and severe acute pregnancy complications, or “near-misses”, their addition remains aspirational. In 2014, the numbers of both stillbirths (approximately 35,000) and neonatal deaths (approximately 34,000) were substantially higher than the number of maternal deaths (approximately 8,000).^19^^,^^20^ It is likely that notification of these deaths may not be possible or complete. A review of perinatal deaths will need an agreed-upon approach to start at facility level and select a subset of cases for review, or to limit the review to cases that are most likely to be preventable, as set out in the new WHO stillbirth and neonatal review guidelines.^21^ In addition, capacity will need to be developed to apply the recently developed cause classification of perinatal deaths (ICD-PM).^22^

In Kenya, a systematic approach was taken to organize structures for sustainability through centrally coordinated confidential enquiry developed and led by a national MPDSR committee and a national MPDSR secretariat embedded within the MOH, and supported by the robust training of a multidisciplinary team of assessors who performed reviews of anonymized case notes. The inauguration of the national committee, appointed by the Cabinet Secretary for Health and chaired by the Director of Medical Services, was a demonstration of the Government's commitment to improving the quality of maternal and newborn health in Kenya. However, in order to ensure the process continues to evolve into a full national enquiry of all maternal deaths, we identified several areas where improvements could be made (Box 2). These include securing longer-term budget allocation and financial commitment from the MOH, securing a national legal framework for MPDSR, and improving processes at the subnational level, such as capacity to accurately classify cause of death, more efficient case note retrieval, and institutionalization of maternal death review at the county and facility levels.

BOX 2 **Suggested Steps to Facilitate Development of a National Confidential Enquiry Into Maternal Deaths System in Kenya**

- **An adequate legal framework.** This is needed to enforce maternal death notification by law, while also reassuring health care providers that the information obtained as part of the Maternal Death Surveillance and Response (MDSR) process will not be used for litigation.^23^^,^^24^ It is anticipated that a forthcoming national health bill will provide a legal framework for MDSR in Kenya.
- **A clear and systematic way of retrieving files.** This is currently done by a team of centrally based staff, supported by the MOH, who visit health facilities in person to retrieve case notes. While this system works for now, in the long term, facilities will need to be encouraged to take responsibility for routinely sending case notes to the secretariat following a maternal death. A mechanism will also need to be in place to retrieve referral and antenatal care notes for maternal deaths.
- **A more efficient process for anonymizing case notes.** This could include scanning case notes at source, such as at the health-facility level, and emailing scanned copies to a dedicated team, who then use an electronic process to block out relevant details in the notes. Clearly this method has resource implications, but the investment would save time, reduce the need for physical storage space, and, perhaps, improve accuracy of anonymization.
- **Improved quality of data.** More sensitization is needed at the facility level to ensure accurate documentation of care provided to all clients. Maternal and Perinatal Death Surveillance and Response is not yet "institutionalized" as an activity at the county or facility levels, and this will require further training in the process of maternal death review, including the importance of complete and accurate recordkeeping.
- **Improved capacity to accurately identify the underlying cause of death.** Challenges in using the WHO International Statistical Classification of Diseases for Maternal Mortality (ICD-MM) have been reported previously.^25^ The MDSR secretariat trained confidential enquiry into maternal deaths (CEMD) assessors in the ICD-MM classification, and developed, tested, and refined a structured tool—the Kenya maternal deaths assessors form—used to review maternal deaths. Specially designed software (Maternal Mortality Audit System, or MaMAS) mirrors the assessors form and has additional capacity for storing, aggregating, and analyzing data extracted from case notes. The original version used in the Republic of South Africa has been refined during the course of the first CEMD in Kenya.
- **Surveillance of maternal deaths.** In Kenya, surveillance of maternal deaths is limited to notification and review of deaths that occur in a health facility. Some counties identify and review maternal deaths in the community, but as yet there is no formal process in place to do this routinely in all counties.
- **Remedial action.** Improved data quality and completeness of case notes will allow assessors to better formulate specific recommendations for improvement at all levels of the health system. This is not an automatic process; it requires interpretation of the findings of a CEMD and discussion among multiple stakeholders. Lessons can be learned from Malaysia, where a systematic and multisector approach is used to identify remedial actions, to which the MOH responds with targeted budget allocation.^26^
- **Sustained source of funding.** Adequate funding is needed to complement current donor funds and to eventually fund the entire enquiry by 2019. If the MOH can commit to funding the CEMD, this will increase participation and confidence in the system by health care providers and encourage ownership by Kenyan health care professionals and the MOH.

## Supplementary Material

Supplement 1

## Notes

**Peer Reviewed**
