# Supplementary material for: Implementing Maternal Death Surveillance and Response in Kenya: Incremental Progress and Lessons Learned
Source: Glob Health Sci Pract. 2017 Sep 27;5(3):345–54. doi: 10.9745/GHSP-D-17-00130 (PMC5620333; doi:10.9745/GHSP-D-17-00130)
Supplement: Supplement 1 [file GHSP-D-17-00130_index.html]

Supplement to Implementing Maternal Death Surveillance and Response in Kenya: Incremental Progress and Lessons Learned | Global Health: Science and Practice

## Supplemental material

- Text s01, PDF - Text s01, PDF
- Text s02, PDF - Text s02, PDF
