# Supplementary material for: Implementing Maternal Death Surveillance and Response in Kenya: Incremental Progress and Lessons Learned
Source: Glob Health Sci Pract. 2017 Sep 27;5(3):345–54. doi: 10.9745/GHSP-D-17-00130 (PMC5620333; doi:10.9745/GHSP-D-17-00130)
Supplement: Supplement 1 [file 17-00130-Smith-Supplement1.pdf]

MINISTRY OF HEALTH

NATIONAL GUIDELINES FOR MATERNAL & PERINATAL DEATH SURVEILLANCE AND RESPONSE

2016

NATIONAL GUIDELINES
FOR
MATERNAL & PERINATAL DEATH
SURVEILLANCE AND RESPONSE

2016

CONTENTS

ACKNOWLEDGEMENT 4

LIST OF ACRONYMS 5

TARGET USERS 5

EXECUTIVE SUMMARY 6

CHAPTER 1: INTRODUCTION 8

- Background Information 8
- Rationale for Kenya MPDSR Guideline 2015 10
- The Gaps in implementation of MPDSR in Kenya 10
- Guiding principles of MPDSR 11
- Other Issues 11

CHAPTER 2: WHY MPDSR 12

- Purpose of MPDSR 13
- Specific objectives: 13
  - Strategies 14
  - Community-based maternal and perinatal death reviews (verbal autopsy) 14
  - Facility-based maternal and perinatal death reviews 14
  - Confidential enquiries into maternal and perinatal deaths 15
  - Near Miss Morbidity Reviews 15
  - The Response Component of MPDSR 15

CHAPTER 3: STRUCTURE OF MPDSR 17

- Kenya MPDSR Organization 17
- Structure and Activities 17
- Community MPDSR Committee 17
- Facility MPDSR Committee (all facility levels) 20
- Sub-County MPDSR Committee 22
- County MPDSR Committee 23
- National MPDSR Committee 24
- National MPDSR Secretariat 25
- National Assessors 26
- The Response Cycle 26

CHAPTER 4: DATA MANAGEMENT AND M&E FRAMEWORK **28**
M&E for MPDSR 28
Data Quality for MPDSR 28
Data flow and use at various levels of the healthcare system 29
Fig 1. Flow chart for Maternal Death notification and review 30
Table 1: MPDSR reporting tools by service delivery level 31
MPDSR INDICATORS 32
CHAPTER 5: LEGAL CONSIDERATIONS **36**
Identifying the challenges 36
IMPLEMENTATION PLAN **39**
References **40**
Annex 1: List of Contributors **42**

ACKNOWLEDGEMENT

The review of the National Maternal and Perinatal Death Surveillance and Response guidelines involved in-depth consultations with a wide range of stakeholders through literature review, interviews, consultative meetings and reviews of the various drafts of the guidelines.

The Ministry of Health feels greatly indebted to individuals and organizations who contributed in one way or another to this elaborate process. Specifically the Ministry would like to thank Dr. Patrick Amoth - Head of the Division of Family Health (DFH), Dr. Kigen Bartilol - Head of the Reproductive and Maternal Health Services Unit, and Dr. Racheal Nyamai- Head of the Newborn, Child and Adolescent Health Unit, who gave invaluable inputs and support to the review and revision exercise.

We further wish to extend our gratitude to Dr. Wangui Muthigani, Dr. Elizabeth Mgamb, Annie Gituto, Joyce Onyango, Elizabeth Washika, Ruth Wayua and Dr. Dan Okoro for leading the process and working with Professor Edwin. O. Were, the lead consultant, to collect, synthesize and incorporate stakeholders' views at every stage of revising the guidelines. The Ministry of Health would like to further recognize the guidelines review taskforce members of the Maternal and Newborn Health Technical Working Group (MNH TWG) who worked tirelessly with the consultant to ensure success at each stage of the process.

The Ministry of Health also wishes to specifically acknowledge the contributions of the various agencies and institutions that led to the production of the Kenya MPDSR Guidelines 2016. The development of the guidelines received technical contributions from the United Nations Population Fund (UNFPA), the United Nations Children's Fund (UNICEF), World Health Organization (WHO), Kenya Country Office, Department for International Development (DfID) through the Liverpool School of Tropical Medicine (LSTM), United States Agency for International Development (USAID) through Measure Evaluation- PIMA and Maternal and Child Survival program (MCSP) and Kenya Obstetrical and Gynaecological Society (KOGS) and multiple experts from medical training institutions, Ministry of Health and Counties health leaders. Lastly, we acknowledge the technical and financial support provided by UNFPA.

The full list of participants and contributors is found in the Annex.

Ag. Director of Medical Services
Ministry of Health

ACRONYMS

CDH	County Director of Health
CEC	County Executive Committee member
CEMD	Confidential Enquiry into Maternal Deaths
CEPD	Confidential Enquiry into Perinatal Deaths
CHEW	Community Health Extension Worker
CHV	Community Health Volunteer
CRHC	County Reproductive Health Coordinator
CU	Community Unit
DHIS	District Health Information Software
DMS	Director of Medical Services
I/C	In charge
ICD 10	International Classification of Diseases 10
HRIO	Health Records Information Officer
MDG	Millennium Development Goals
MDR	Maternal Death Review
M&E	Monitoring and Evaluation
MMR	Maternal Mortality Rate
MO	Medical Officer
MOH	Medical Officer of Health
MPD	Maternal and Perinatal Death
MPDB	Medical Practitioners and Dentist Board
MPDR	Maternal and Perinatal Death Review
MPDSR	Maternal and Perinatal Death Surveillance and Response
PNM	Perinatal Mortality
RMHSU	Reproductive and Maternal Health Services Unit
SCHRIO	Sub-County Health Records Information Officer
SCRHC	Sub-County Reproductive Health Coordinator
WHO	World Health Organization
WRA	Women in Reproductive Ages

Target Users

The target users of the Kenya MPDSR guidelines 2015 include:

1. National and County Government, Maternal and Neonatal Health (MNH) Program Officers
2. Members of all MPDSR committees at community, facility, sub-county, county and national levels
3. All service providers in MNH
4. Training institutions involved in MNH
5. Professional associations with interest in MNH
6. Implementing partners involved in MNH in Kenya
7. Development Partners involved in MNH in Kenya

EXECUTIVE SUMMARY

The global maternal mortality ratio decreased by about 44% over the last 25 years from 385 to 216 per 100,000 live births. Similarly, the annual maternal deaths also dropped by 43% over the same period from 532,000 in 1990 to 303,000. However, this masks the true picture of the burden. Developing countries account for 99% of the maternal deaths; with sub-saharan Africa accounting for 66% (201,000 annual deaths). Perinatal mortality also follows the same regional pattern as maternal mortality. It is estimated that over 3.3 million stillbirths occur every year and 4 million neonatal deaths of whom 90% succumb within the first 7 days of life.

Kenya is among the top 10 countries in the world with highest burden of maternal mortality, losing between 5000 to 6000 mothers every year due to pregnancy and birth complications. Although Kenya's MMR has reduced from over 590 in the 90's to 362 per 100,000 in 2014 (KDHS 2014), Kenya did not attain the MDG 5 by end of 2015. According to the KDHS 2014, this change has been observed as within the statistical margin hence insignificant reduction.

The health information system and the Civil Registration and Vital Statistics System (CRVSS) have not adequately captured the magnitude of maternal deaths as only 15% of the maternal deaths are recorded in the Department of Health Information System (DHIS) and only 60% of all deaths are captured by the CRVSS. The perinatal mortality is also not adequately captured by the CRVSS. Inadequate measurement of maternal and perinatal deaths contributes to a lack of accountability and in turn to a lack of progress. Strengthening the monitoring and evaluation system especially data quality is key. The data and information generated by MPDSR will be linked to the already existing platforms of surveillance such as Integrated Disease Surveillance and Response which has proved to be reliable. Innovative use of technology e.g. cell phone to track maternal and perinatal death will enhance the system operations.

Concerted multi-sectoral efforts must be put in place to eliminate maternal mortality especially when evidence shows that nearly 80% of the causes of maternal mortality are preventable through high impact cost effective interventions even in low resource settings. The leading causes of maternal deaths are haemorrhage, hypertension complications in pregnancy and sepsis; while the causes of newborn deaths are related to asphyxia, prematurity and sepsis.

It is for these reasons that Kenya has developed this Maternal and Perinatal Death Surveillance and Response (MPDSR) Guidelines which has been adapted from the WHO MDSR Technical Guidance. While WHO is scheduled to release the technical guidance on perinatal mortality, Kenya took a step ahead to integrate tracking and review of perinatal deaths in this guideline.

The national MPDSR guideline is aligned to the National Health Policy 2013 – 2030 as well as the National Health Strategy and Investment Plan 2013 – 2018, where reduction of maternal and neonatal mortalities have been prioritized. The review has also been informed by the implementation of the new governance structure as spelt out in the Constitution 2010. It also draws its relevance from other global initiatives such as UN Secretary's Global Strategy on Every Woman Every Child and Adolescent health and Sustainable Development Goals which calls for accountability for maternal, newborn child and adolescent health. These have also put emphasis on importance of strengthening civil registration and vital statistics.

Development of the MPDSR guideline has been rigorous and was done in a consultative manner. Key stakeholders among them government departments (national and county levels) UN agencies, non-governmental organizations, and civil society organizations among others have contributed.

The purpose of this document is to provide guidance on how to conduct Maternal and Perinatal Death Reviews (MPDRs) and near miss reviews in the community and the health facilities. It further guides the approach to surveillance of maternal and perinatal deaths, the reporting pathways and how sustainable response to address avoidable causes of death is incorporated. In addition, it creates mechanism for conducting confidential enquiry to maternal and perinatal deaths, which has never been present in the country.

This MPDSR guideline covers the following areas:

- Introduction of MPDSR highlighting the burden of maternal and perinatal mortality as well as why MPDSR is important.
- The governance structure and processes of MPDSR including response cycle.
- Data management, monitoring and evaluation
- Legal considerations in implementing MPDSR.

CHAPTER 1: INTRODUCTION

Background Information

An estimated 289 000 women worldwide died from pregnancy and its complications in 2013. Africa and especially sub-Sahara Region has the highest burden of maternal and perinatal mortalities. The maternal mortality ratio in Africa is estimated to be 500/100,000 live births (WHO, 2015). Reported maternal mortality underestimates the true magnitude by up to 30% worldwide and by 70% in some countries (3, 4).

Kenya's maternal mortality ratio (MMR) and perinatal mortality rate still remains high at 362 per 100,000 and 28/1000 live births respectively. Wide disparities in MMR have been noted between the counties and ranges between 3,790/100,000 live births and 187 /100,000 live births for the county with the highest and lowest MMR respectively (NCPD, 2013). The disparities in socio-economic and cultural factors in various regions in the country have resulted in variable maternal mortality ratio (MMR) estimates. Late or inconsistent attendance at antenatal care (ANC) clinics, variable quality of antenatal care, and poor access to skilled care during pregnancy, delivery and the postpartum period contribute to adverse outcomes for mother and baby.

The major causes of maternal mortality in Kenya, like in most parts of the world are haemorrhage, complications from hypertensive disease in pregnancy, sepsis and abortion complications. HIV related deaths (20%) comprise the largest proportion among the indirect causes of maternal mortality. Up to 53% of perinatal deaths are intra-partum and therefore avoidable through quality management of labour. Evidence shows that most of the causes of maternal and neonatal deaths can be averted with early access to quality emergency obstetric and new-born care.

In order to eliminate preventable maternal and perinatal deaths several measures have been taken by the Ministry of Health. They include scaling up of Emergency Obstetrics and New-born Care training countrywide, Free Maternity Services Initiative, the Beyond Zero campaign and instituting Maternal and Perinatal Death Surveillance and Response (MPDSR) mechanisms among others.

The health information system and the Civil Registration and Vital Statistics System (CRVSS) have not adequately captured the magnitude of maternal deaths as only 15% of the maternal deaths are recorded in the District Health Information Software (DHIS) and only 60% of all deaths are captured by the CRVSS. Inadequate measurement of maternal deaths contributes to a lack of accountability and in turn to a lack of progress.

Every maternal and perinatal deaths should be accounted for by systematic tracking and investigating the contributing factors. In addition, recommendations on how these factors can be addressed should be made. Maternal and Perinatal Death Surveillance and Response (MPDSR) is a model of such a

system. By investigating maternal and perinatal deaths, MPDSR inherently places value on their lives – an important mechanism of accountability for families, communities and the Nation (5). MPDSR is a system of confidentially auditing all maternal and perinatal mortalities and near misses and strategically using this information to guide public health decisions, actions and monitoring their impact. This is achieved by strengthening of data collection, use and the introduction of quality improvement through Monitoring and Evaluation (M&E) in Health Services. A maternal death surveillance and response system that includes maternal death identification, reporting, review and response can provide the essential information to stimulate and guide actions to prevent future maternal deaths and improve the measure of maternal health.

In 2004, WHO (8, 9) provided a comprehensive description of approaches to maternal death audit including: Facility Based Maternal Death Audit, Community Based Maternal Death Audit - employing Verbal Autopsy methodology. To complement Maternal Death Audit, and recognizing the relative rarity of maternal mortality, methodology for Near-Miss Maternal Morbidity Audit was developed. It has been observed that these approaches are only effective if a response component is built into the system to ensure that all preventable deaths are indeed prevented in the future through mechanisms for continuous quality improvement. To this effect, WHO also described the Clinical Audit that should enable facilities to continuously examine their activities and respond in a way to prevent avoidable deaths in the future.

The Ministry of Health developed maternal death review guidelines in 2009 and subsequently gazetted maternal death as a notifiable event. In spite of the structures established to notify and audit maternal and perinatal deaths, there was a lack of systematic approach in reporting and reviewing of the maternal and perinatal deaths. More recently, and building on the guidance in Beyond the Numbers(8), WHO has provided Maternal Death Surveillance and Response (MDSR) technical guidelines(10) for the systematic national adoption. The first step in the MDSR is that maternal death must be a notifiable event and incorporated into Integrated Disease Surveillance and Response. An additional "cultural" requirement is that maternal death reporting must be done at specified time periods even when there is no death to report, giving rise to the concept of "zero reporting". Critical to MPDSR, is maternal and perinatal death identification, accurate classification per ICD-10 guidelines (11).

As part of this process, strengthening of health information systems and adoption of emerging technologies such as mobile phone applications report and track pregnancies, and mothers through postnatal period may be important implementation tools for nations. Rwanda, for example has adopted mobile phone technology for this purpose (12). Another issue critical to the success of MPDSR implementation is the deliberate creation of an environment of "no blame" in the maternal death review process. Service providers and relatives involved in the care of cases that end in maternal death must be reassured, that the proceedings of a maternal and perinatal death review will be anonymised and cannot be used in any litigation procedures against them.

Phased implementation of such guidelines, depending available national resources and political commitment is recommended. The facility based maternal death audit can be rolled out first and when

well established, incorporate community based audits, graduating confidential enquiry into a sample of maternal deaths and eventually ending with a national confidential enquiry into all maternal deaths. On another level, counties and facilities can begin by having only maternal death reviews (MDR) and when established, add perinatal death review (MPDSR).

The MPDSR guidelines are well grounded in Kenya Health Policy, Reproductive Health Policy (14), Maternal and New-born Health Implementation plan, Vision 2030(15) and even the Constitution (16). Consequently, based on national consensus on the national adaptation of MPDSR and the prevailing political will in the implementation of the same guidelines, Kenya is primed to achieve the maternal and neonatal health targets consistent with Vision 2030. There has been progress in Kenya MPDSR such as incorporation of the MPDSR indicators in the DHIS and training of the county health care workers. (17).

In several countries, the introduction of maternal and perinatal death review processes have resulted in improved quality of maternal and perinatal services and consequently, a reduction in maternal mortality, notably in South Africa, where maternal death review processes were introduced in 1998.

The purpose of this document is to provide guidance on how to conduct Maternal and Perinatal Death Notification and Reviews (MPDRs) as well as near miss reviews in the community and the health facilities. It further guides the approach to surveillance of maternal and perinatal deaths, the reporting pathways and how sustainable response to address avoidable causes of death is incorporated.

Rationale for Kenya MPDSR Guideline 2015

The current effort to revise the Maternal Death Review guidelines of 2009 has been informed by several reasons:

1. Maternal and perinatal mortality remain a major burden in Kenya and requires a structured multi-sectoral response.
2. The guidelines needed to be congruent with and incorporate the new devolved structure of Governance, following the implementation of The Kenya Constitution 2010.
3. The national maternal perinatal death and review guidelines needed alignment to the Revised WHO MDSR Technical Guidance by strengthening the Response component.
4. The transition from MDGs to SDGs.

Gaps in implementation of MPDSR in Kenya

- Weak Data system.
    - o Inadequate data collection tools
    - o Inadequate capacity to fill in appropriate data
- Weak feedback system from higher levels.
- Weak system to address possible litigation hence fear of reporting/auditing maternal deaths.
- Lack of clear system of orientation, accountability and, leadership.
- Lack of regulatory and professional requirement to report MPDs.

- No national annual report to inform advocacy for resource allocation for implementation of action plans. Timing of the presentation of annual report should be synchronized with the budget cycle.
- Inadequate appropriate response to recommendations leads to burn out when same issues are discussed in every MDR meetings.
- Existence of parallel systems of review: Perinatal deaths discussed by paediatricians separate from the maternal death reviews – no joint efforts. There are no tools to capture stillbirths and early perinatal deaths need to be developed and disseminated.

Guiding principles of MPDSR

- No blame policy.
- Death reviews focus on health systems not individuals.
- MPDSR meeting is primarily an educational experience for all participants.
- In MPDSR programs, a zero-reporting principle is adopted, meaning that reports are made regularly even if no death has occurred.
- Documentation of patient case notes is the main source of information for the MPDSR process.
- Relatives are the chief source of information for verbal autopsy. Should be approached after a culturally appropriate duration of mourning.
- Death audit data are anonymised and CANNOT be used for disciplinary purposes.
- MPDSR to be incorporated into curricula of medical training institutions.
- The death reviews are incomplete without response to prevent avoidable factors in the future.
- The response mechanism involves a multi-sectorial approach.

Other Issues

Early pregnancy identification

- Identification of early pregnancies is critical to full documentation of maternal mortality especially those related to abortion and ectopic pregnancies.
- An interim approach is to work with the chiefs to ensure all deaths of women in the reproductive age (WRAs) are audited for possibility of pregnancy especially for those where there is genital bleeding as part of the presenting symptoms.
- ICD -10 training needed and should include sensitization of community based MPDSR personnel.

CHAPTER 2: WHY MPDSR

In Kenya approximately 6000 women die due to child birth related complications every year; and there has only been an 18% reduction in MMR in the past 20 years.

To accelerate progress, the Secretary-General of the United Nations re-launched the Global Strategy for Women's and Children's Health in September 2015. The Global strategy targets ending preventable maternal, new-born and child deaths as intervention to attaining sustainable development goal (SDG 3). It also emphasizes the strengthening of civil registration and vital statistics to ensure that deaths and their causes can be registered and monitored.

MPDSR is a strategy to document the burden of maternal and perinatal deaths, the causes and avoidable factors coupled with a clear response plan to avoid future deaths and make significant contributions to the achievement of the recently launched Sustainable Development Goals (SDGs). Specifically, MPDSR responds to SDG 3: Ensure healthy lives and promote well-being for all at all ages[1]. The relevant targets for this goal are:

- By 2030, reduce the global maternal mortality ratio to less than 70 per 100,000 live births.
- By 2030, end preventable deaths of newborns and children under 5 years of age, with all countries aiming to reduce neonatal mortality to at least as low as 12 per 1,000 live births and under-5 mortality to at least as low as 25 per 1,000 live births.

Kenya has demonstrated a strong political will to improve the quality of care for pregnant women and women in the post-natal period. In 2004 maternal death notification was made mandatory (through government circular) and free maternal health services were introduced in 2013. Increase in access to skilled maternal care as a result, has increased the need for quality maternal health services[2].

A review of all maternal deaths notified and reviewed nationally between 2004 and 2006 revealed under reporting of maternal deaths (Only 46% of all maternal deaths reported via HMIS were notified). Furthermore only 56.8% of all maternal deaths were notified within 24 hours of death and 30% of all maternal deaths were notified after 1 week. This review showed both under reporting and poor compliance to the 2004 Ministry of Health circular. The recommendations after the review were, (i) the need to train health workers on how to complete the forms and (ii) a professional should be nominated to head and oversee the MDR process at each health facility (unpublished report Kenya Essential Health Services, 2006).

---

[1] http://www.un.org/sustainabledevelopment/health/ accessed on September 11, 2015.

[2] Bourbonnais, N. 2013. Implementing free maternal health care in Kenya, Challenges, strategies and recommendations. Kenya commission on Human rights. www.knchr.org

The Kenya government re-launched the facility based maternal death audits (MDA) in August 2009, new guidelines were developed which re-emphasised issues of accountability on the part of the health workers that was hitherto stressed when the MDA was introduced in 2004. The new guideline focused more on correcting health system problems rather than individual faults. New forms were developed, a notification form and a more detailed review form for the MDA process was introduced[3] (MOPHS, 2009).

A similar review conducted in 2011 for maternal deaths that occurred between 2008 and 2010, revealed that only 20% (433) of 2203 maternal deaths recorded through the HMIS were reviewed at health care facilities. Also 25% of maternal review forms were poorly completed and there was no evidence of response to recommendations from MDRs implemented at health care facilities or at national level.

Following the publishing of the WHO technical guidelines for MPDSR in 2013, Kenya included the maternal deaths as a notifiable event through the district health information software 2 (DHIS 2). Orientation workshops were conducted and used to train health care workers at all levels. A review of the system done in 2014 has provided mixed results, the system appears to be working under partner funded programmes within few counties. Overall notification and reporting of maternal deaths has not improved.

Causes of maternal and perinatal deaths are poorly documented and rarely are post mortems done when these deaths occur. There are few pathologists, much fewer ICD coders in Kenya, post mortem examinations are not routinely conducted by pathologists and recent evidence suggest that most causes of maternal deaths arrived at through facility based MDRs are inaccurate[4].

Purpose of MPDSR

The main purpose of MPDSR is to take actions to eliminate preventable maternal and perinatal deaths.

Specific objectives

- To document the burden of maternal and perinatal deaths.
- To gain understanding of the health system failures that led to the maternal/ perinatal death or complication.
- To raise awareness among health professionals, administrators, programme managers, policy makers and community members about those factors in the facilities and the communities which, if avoided, the death may not have occurred (the avoidable factors).
- To stimulate action to address the avoidable factors thereby prevent future maternal and perinatal deaths.

---

[1] Kenya Ministry of Health and Public Sanitation 2009. Maternal death Review Guidelines.

[2] Owolabi H, Ameh CA, Bar-Zeev S, Adaji S, Kachale F, van den Broek N (2014) Establishing cause of maternal death in Malawi via facility-based review and application of the ICD-MM classification BJOG (121) doi: 1111/1471-0528.12988.

Strategies

There are several approaches that can be used to monitor maternal and perinatal deaths and clinical practice. All have the objective of reducing maternal and neonatal mortality and morbidity by improving the quality of MNH care provided.

Approaches that are proposed for use in Kenya guidelines include:

- Community-based maternal and perinatal death reviews (verbal autopsy).
- Facility-based maternal and perinatal death reviews.
- Confidential enquiries into maternal deaths.
- Near miss reviews.

Community-based maternal and perinatal death reviews (verbal autopsy)

Community-based maternal death review is a method of finding out the medical causes of death and ascertaining the personal, family or community factors that may have contributed to the deaths in women who died inside or outside of a health facility. The entry point is to ascertain from the immediate family if the deceased woman was pregnant or not. Subsequently, questions are asked about the major symptoms and presentations of the most common direct causes of mortality i.e. haemorrhage, infection, hypertensive disease, obstructed labour and abortions. Indirect causes of maternal death are also similarly enquired about. Once the presenting features of the main illness that led to the woman's death are identified, the cause of death is usually assigned following International Classification of Diseases 10 (ICD 10) for wider comparison and ease of aggregation of data at the county or national level. Efforts are also made to identify causes of delay in accessing MNH services: focusing on any causes in delay in recognizing the need to go to a health facility (1st delay factors), delay in actually arriving at the facility once the decision to go to facility was made (2nd delay factors).

For perinatal deaths, verbal autopsy revolves around whether there was a still birth or baby died within 7 days after being born alive. Maternal conditions during the pregnancy are then enquired about. Also important is the enquiry about the symptoms the new-born exhibited before death.

Facility-based maternal and perinatal death reviews

A facility-based MDR or PDR is a qualitative in-depth investigation of the causes of, and circumstances surrounding maternal/ perinatal deaths occurring at health facilities. It is particularly concerned with tracing the path of women who died, through the health care system and within the facility, to identify any avoidable remediable factors that could be changed to improve maternal care in the future. Deaths are initially identified at the facility level but such reviews are also concerned with identifying the combination of factors at the facility that may have led to the delay in receiving quality MNH services (3rd delay factors) and in the community that contributed to the death, and which ones were avoidable (1st and 2nd delay factors).

Similar to MDR, perinatal death reviews involves the maternal health providers but also the new-born care team. It focuses on identifying maternal conditions during pregnancy and around delivery and the care provided to the neonate up to 7 days after delivery. Avoidable causes are then prioritized for remedial action.

No maternal or perinatal death review is complete unless it is linked with an attempt to respond to the findings with appropriate action.

Confidential enquiries into maternal and perinatal deaths

Confidential enquiry into maternal deaths is a systematic multi-disciplinary anonymous investigation of all or a representative sample of maternal deaths occurring at an area, sub-county, county, or national level. It identifies the numbers, causes and avoidable or remediable factors associated with them. Confidential enquiries validate the national aggregated data on death reviews from individual, community and facility MPDSR committees. It involves fresh interviews with persons involved in the care of the woman at the time of death including her family and her health providers, fresh assessment of source documents including patient's notes by independent assessors and discussions to reach consensus on the cause of deaths enquired into.

Near Miss Morbidity Reviews

"The identification and assessment of cases in which pregnant women survive obstetric complications. There is no universally applicable definition for such cases and it is important that the definition used in any survey be appropriate to local circumstances to enable local improvements in maternal care." However the WHO defines maternal near-miss (MNM) as a woman who nearly died but survived a complication that occurred during pregnancy, childbirth or within 42 days of termination of pregnancy (WHO 2011).

In this guideline, this strategy has been adopted as a routine part of MPDSR. The near-miss approach yields results that inform policy decisions for improving the quality of maternal health care in individual health-care facilities.

The Response Component of MPDSR

Maternal and or perinatal death review needs to be seamlessly connected to a response strategy. This involves ensuring that action points to prevent avoidable deaths are implemented at community, facility, sub-county, county and national levels.

At the ***Community level***, response entails feedback to the community, partnering to alleviate multiple causes of first delay in accessing care through discussion forums such as community dialogue days and barazas and development of locally relevant community based transport networks.

At ***Facility level***, response largely focuses on addressing causes of the third delay. A tried and tested strategy for this is clinical audit and or other continuous quality improvement programs.

At ***Sub-County level***, response entails aggregating community and facility data and addressing broad and common avoidable factors that affect multiple communities and or facilities. Sub-county level response should be escalated to the level of the county if common avoidable factors affect several sub-counties.

At ***County level*** the response should encompass monitoring of the MPDSR activities countywide. This should point out the sub-counties, facilities and or communities that report heavy burden of deaths/ MPDs for more intensive scrutiny and response in addressing countywide avoidable causes of death. The other component of response at the county level is reviewing relevant county specific multi-sectoral frameworks and aligning them to achievement of MPDSR goals and also allocating necessary resources to support response as guided by the County MPDSR reports.

Finally, at ***National level***, there should be national oversight of the complete MPDSR program through close monitoring of the MPDSR indicators to identify high burden counties as a basis for resource allocation for focused response and technical support. Again, review of relevant legislation and resource mobilization to address national avoidable factors where the need is greatest is a critical response parameter at this level.

CHAPTER 3: STRUCTURE OF MPDSR

Kenya MPDSR Organization

The model is grounded in an evidence-based premise that improving quality of maternal care will result in a reduction in maternal and perinatal mortality. The model seeks to institutionalize MPDSR at both community and facility levels with the aim of identifying underlying causes of maternal deaths that can be addressed locally. The response component is critical to the completion of the loop and will often involve actions that may or may not require additional resources to achieve. Continuous quality improvement and strengthening of the health systems through clinical audit is a proven strategy for implementing response to maternal and perinatal deaths.

Emphasis has been placed on simplicity of surveillance, reporting through the DHIS platform, to multiple levels simultaneously to ensure wide availability of data at various levels culminating in presentations of the County data to the County Assembly Committee on Health and National data to the Joint Senate & Parliamentary Committee on Health. This approach is meant to unlock resources at the two levels of government for the response component. The model also recognizes the current role of the Assistant Chief and Chief (under Ministry of Interior) in registration of Births and Deaths and seamlessly incorporates them in the MPDSR process.

The model entrenches perinatal death reviews and reporting at all levels in the MPDSR and concomitantly simplifies the tools for data collection at each stage. The overarching principle in development of data collection tools is brevity and contribution to the documentation of: the burden of the maternal and perinatal deaths, who dies, where they die, why they die and what can be done to prevent similar deaths in the future. Data that does not directly address these five questions is considered burdensome to the system and has been omitted in favour of brevity. The guidelines envisage that where additional in-depth data is required, the assessors carrying out CEMD (or CEPD) will review all source documents to access such data.

Structure and Activities

In Kenya, a 5-tiered committee structure will be established: Community, Facility, Sub-county, County and National committees. Each committee is guided on the core membership and leadership but retains the prerogative to co-opt other members as the committee leadership sees fit to facilitate the functions of each committee for each death reviewed.

Community MPDSR Committee

Core Members:

1. Assistant Chief-Chairperson
2. CHEW- secretary
3. Clinician from the link facility
4. CHV from the community unit where the deceased comes from

5. Chair of Community Health Committee
6. Community midwife (if available).

Co-opted Members:

Civil Society groups

Leadership

Chaired by Assistant Chief – the PS, through the Office of Interior and Coordination will request the County Commissioner to appoint; there will also be communication to the county Directors of Health. Co-opts other members as needed

Terms of Reference

1. Notify all deaths of Women of Reproductive age (WRA) as well as deaths of adolescents 10-14 years occurring in the specific community.
2. Hold death review (verbal autopsy) to determine the causes of maternal deaths occurring within the community.
3. Provide a monthly report on maternal and perinatal deaths in the community.
4. Implement community based activities to prevent occurrence of similar deaths in the future. Such activities shall be identified and agreed upon during the death review meetings.
5. Advocacy meetings at the community to promote maternal and new-born health.

Activities

Maternal Death Notification

All deaths of women in the reproductive ages (WRA) will be reported by the CHV within 24 hours. For these deaths, it may not be immediately obvious if the woman was pregnant or not and hence to avoid missing cases of maternal death, it is recommended that all deaths occurring in the community involving WRA be reported. If the death occurs in the facility, the pregnancy status can be determined from the history or any relevant investigations and only maternal deaths shall be notified. Perinatal deaths will also be notified and reported monthly.

1. Immediately a WRA or adolescents 10-14 years dies at home, within the community, the CHV notifies the CHEW in person, by call or text message.
2. The CHEW uses the line list to document all deaths of WRA reported including adolescents 10-14yrs and uses a screening tool to determine if this was a maternal death.
3. The CHEW then informs the SCRHC by telephone call or short message service of the death immediately they confirm that it is a Maternal Death.
4. The CHEW completes and files the Death Notification Form with the link facility in charge and immediately submit it to the facility HRIO/ SCRIO for uploading into the DHIS.
5. The facility/ sub-county HRIO completes the process by uploading the information into the DHIS database. The whole process from the CHV to the DHIS must be completed within 24 hours.

Maternal Death Review

The Community MPDSR committee will meet within one month of the death to carry out a verbal autopsy on the death. In setting the date for the verbal autopsy, due consideration must be made for the cultural sensitivities about the burial and post burial rites. The key sources of information will be the persons who were present when the woman became unwell, when she died, the husband and a female relative and, where the pregnancy has ended, persons who were present during her delivery. The CHEW invites all persons with information about the death to a verbal autopsy meeting within 14 days of the maternal death.

1. The CHEW together with the CHV visits the home of the deceased to obtain information about the death.
2. During the verbal autopsy, the CHEW uses the Verbal Autopsy Form as a guide to obtain maximum information.
3. After obtaining the information, the CHEW liaises with the assistant chief to invite all the members of the community MPDSR committee with information about the death to a verbal autopsy meeting.
4. The meeting begins by determining if the woman was probably or definitely pregnant or within 42 days after delivery or termination of the pregnancy. The Verbal Autopsy Form guides the documentation process. During the documentation, three names of the deceased will be included.
5. The CHEW submits the findings of the verbal autopsy to the link facility MPDRS committee for review and to gives feedback to community.
6. The CHEW submits Verbal Autopsy form to the chair of the link Facility in charge who forwards it to Sub-county RH Coordinator and facility HRIO/SCHRIO for uploading into the DHIS2 platform.

Perinatal Death Notification and Reviews

1. The CHV informs the CHEW of a perinatal death immediately.
2. The CHEW completes a Perinatal Death Notification Form obtaining the information from the mother or the immediate relatives of the mother of the dead baby within 72hours of the perinatal death.
3. The CHEW files the notification and also keeps a line list of all perinatal deaths.
4. The CHEW submits copies to the link facility in charge who shall report the community perinatal deaths together with those occurring at the facility to the SCRHC and the Facility HRIO/SCHRIO for uploading into the DHIS and onward transmission to higher committees.
5. The link facility compiles a monthly report on perinatal deaths by the 5th of every month.

Response at the Community

1. Findings and recommendations of maternal death review are shared with the community during community dialogue days or during Barazas.
2. The action points on perinatal deaths are similarly discussed and implemented.
3. Any feedback from the Facility MPDSR Committee is discussed at the same forums.

Facility MPDSR Committee (all facility levels)

Core Members:

1. Medical Superintendent / Facility in charge - Chair
2. Head of Quality/Work Improvement Team
3. Maternity in charge- Secretary
4. MCH Representative
5. Obstetrician
6. Paediatrician or representative from new-born unit or paediatric ward depending on the hospital set up
7. Health Records Information Officer (HRIO) responsible for uploading into the DHIS(Facility HRIO or SCHRIO)
8. Reproductive Health Coordinator
9. Health Administrator
10. Anaesthetist per level
11. Representative of training institution if students are attached to the facility

Note: The sub-county Reproductive Health (RH) Coordinator will be part of the MPDSR team for lower level health facilities.

Leadership

Chaired by the Head of the Facility – appointed by the County Director of Health (CDH)
Co-opts other members as needed

Terms of Reference

1. Hold monthly meetings over and above maternal and perinatal death review meeting.
2. Notifies all maternal deaths occurring within the facility.
3. Hold death review meetings to determine the cause of death of all maternal deaths occurring within the facility.
4. Provide a monthly report on maternal and perinatal deaths in the facility.
5. Implement institutional activities to prevent occurrence of similar deaths in the future. Such activities shall be identified and agreed upon during the death review meetings.
6. Provide feedback to the community MPDSR committee as needed.

Activities

Maternal Death Notification

1. All deaths involving WRA within all facilities shall be evaluated for possibility of pregnancy and hence being maternal death.
2. Immediately a death of a WRA occurs in any unit / ward of a facility, the unit in charge, informs the Facility in charge of the death and indicates if the WRA was pregnant or had a pregnancy that ended within 42 days of delivery.
3. If the WRA was pregnant or had a pregnancy that ended within 42 days of delivery, the unit in

charge completes the Death Notification Form and submit it to the facility HRIO for uploading into DHIS within 24 hours, and also informs the SCRHC by sms or call.

4. Each facility is required to submit a monthly report on the number of maternal deaths (both at the facility and community levels). This includes zero reporting is no maternal death was reported during the month. The number can be obtained by counting the maternal death notification forms filed.
5. The facility sends weekly reports of the number of maternal deaths reported (both at the facility and community) together with the weekly reportable priority diseases/events to the sub-county disease surveillance officer as part of the IDSR. This includes zero reporting if there were no maternal deaths in that epidemiologic week.

Maternal Death Reviews

1. The head of the unit/ward where the woman was admitted when she died initiates the maternal death review process, if the woman was known to be pregnant or within 42 of delivery or termination of the pregnancy.
2. The unit in-charge summarizes the history and relevant findings about the clinical presentation, investigations, treatment and circumstances surrounding the death.
3. The Facility MPDSR committee meets within 7 days of the death to discuss the case and complete the Maternal Death Review Form.
4. A designated note taker documents all the key findings and decisions taken including the action plans developed.
5. No names for service providers are included in the summary document. All summary documents shall be kept under the sole custody of the facility Health Records Information Officer or facility in-charge (where there is no facility HRIO) after the meeting and shall not be used for either disciplinary or litigation purposes.
6. For near miss reviews, the same process is followed as for MDR but the surviving patient will be a key source of information used at the meeting. The unit in charge obtains relevant information from the survivor before the MPDSR committee meeting.
7. Facility MPDSR committee decides to collect additional data where necessary.
8. The committee lead oversees the compilation of a report summarising each maternal death and or the near miss morbidities with primary and secondary causes of death and possible avoidable factors and submits it to the Sub-county MPDSR committee through the SCRHC.
9. The monthly MPDSR report will have a report on all the maternal deaths recorded during the month or zero reporting, if none was recorded.

Perinatal Death Notification and Reviews

1. The facility /unit in-charge completes the Perinatal Death Notification Form for all perinatal deaths at the facility and keeps a line list of all the perinatal deaths.
2. The perinatal death notification form is then submitted to the facility/Sub-county HRIO for uploading into the DHIS.
3. The facility /unit in-charge convenes a monthly MPDSR committee meeting to review all the perinatal deaths occurring in the facility.

4. The perinatal death review forms completed will be submitted to the facility/ sub-county HRIO for uploading into the DHIS 2 SCRHC.

Response at the Facility

1. Develops action plans to avoid future preventable maternal deaths, perinatal deaths and near miss morbidities. Action plans based on simple institutional quality improvement activities.
2. Identifies person(s) responsible for implementing action points.
3. Reviews progress in implementing action plans at the next MPDSR meeting.
4. Develops and delivers feedback information for the relevant levels –Sub-County MPDSR Committee, Community MPDR committee, and the community regularly.

Sub-County MPDSR Committee

Core Members:

1. Sub-County Medical Officer of Health-Chair
2. Sub-County Disease Surveillance Coordinator
3. Sub-County Public Health Nurse
4. Sub-County Health Records Officer
5. Sub-County Reproductive Health Coordinator - Secretary
6. Sub-County Lab i/C
7. Sub-County Health Administrator
8. Sub-County Community Strategy focal person

Leadership

Sub-county MOH - Chairs. Co-opts other members as needed.

Terms of Reference

1. Monitor functionality of MPDSR system in the sub-county.
2. Develop a quarterly sub-county MPDSR report aggregating all community and facility maternal and perinatal deaths.
3. Identify communities / facilities reporting high burden of M&PDs for locally relevant action.
4. Provide feedback to the community and facility MPDSR committees under jurisdiction.
5. Support the lower level health facilities and community MPDSR committees to conduct their maternal and perinatal deaths reviews.

Activities

1. Sub-county MPDSR committee lead coordinates quarterly meetings to review MPDSR reports from all facility and community based MPDR committees.
2. Synthesises the data and compiles a sub-county quarterly report for submission to county level.
3. Records proceedings of all meetings conducted.
4. Provides feedback to all facility and community MPDSR committees.
5. Provides support (technical and other resources) to all facilities and communities to implement the recommendations / action points.

6. Monitors MPDRS processes in the sub-county, ensure they are all functional and track sub-county specific MPDSR indicators through the dashboard.

County MPDSR Committee

Core Members:

1. County Director of Health
2. County Chief Nursing Officer
3. County Reproductive Health Coordinator
4. County Health Records and Health Information Officer
5. Community Strategy Focal Person
6. County Disease Surveillance Focal Person
7. Representative of training institutions
8. County Health Administrator.
9. County Civil Registrar
10. Representative of Mission and Private Health Facilities

Leadership

Chaired by County Director of Health appointed by the CEC Health; co-opts other members as needed.

Terms of Reference

1. Provides oversight role and technical support to ensure institutionalization of MPDSR system in the county.
2. Develop a quarterly county MPDSR report aggregating all sub-county MPDSR reports.
3. Identify sub-counties reporting high burden of Maternal and Perinatal deaths Ds for locally relevant action.
4. Prepare six monthly county MPDSR report to be presented to the county Assembly Committee on Health by the County Executive Committee Member (CEC), Health.

Activities

Works through a secretariat based at the office of the CDH. The secretariat consisting of the CRHC, the CHRIO, County Community Strategy Focal Person, County Disease Surveillance Officer, Obstetrician and an administrative secretary:

1. Receives and reviews MPDSR reports from all Sub-County MPDSR committees and cross-checks against the DHIS 2 data.
2. Synthesises the data and compiles a County six monthly MPDSR report, from the Sub-County reports, for submission to CEC and the Governor's office.
3. Submits a copy of their MPDSR report to the National MPDSR committee after validation by the CEC.
4. The County report shall (but not limited to):

    a. Present the burden of maternal and perinatal death (in absolute numbers) and disaggregated by sub-counties

b. Calculate the perinatal mortality rate disaggregated by sub-counties

c. Present the causes of maternal and perinatal deaths in the county

d. Calculate the obstetric case fatality rate (%) for the counties

e. Aggregate the avoidable factors to be addressed at the county and sub-county level, to prevent maternal and perinatal deaths.

f. Any near-miss cases (and their diagnoses)

g. Identify opportunities for a multi-sectoral response within the county.

5. Provides feedback to all Sub-county MPDSR committees.
6. Provide support (technical and other resources) to all sub-counties and facilities and communities to implement the recommendations / action points.
7. Monitor MPDRS processes in the county, ensure they are all functional and track county specific MPDSR indicators through the dashboard.

At the County MPSDR Committee:

1. Meets quarterly.
2. Records proceedings of all meetings conducted.
3. Receives, considers and adopts the quarterly County MPDSR aggregated data from the secretariat.
4. Organizes county forum for discussing county aggregated data for quality improvement, sensitization and community awareness.
5. Presents the County aggregated data to the County Assembly Committee on Health.
6. Conduct county advocacy forums. Copies the senate for information and lobbying for resources.

National MPDSR Committee

Core Members:

1. Director of Medical Services
2. Head, Department of Preventive and Promotive Health Services
3. Head, Division of Family Health
4. Ministry of Health, Legal Advisor
5. Director Civil Registration Services
6. Representatives of Professional organizations (Kenya Obstetrical and Gynaecological Society, Kenya Paediatric Association and Kenya Medical Association)
7. Representatives of training institutions (College of Health Sciences, University of Nairobi, Chair OB/GYN Department)
8. Regulatory Bodies (Nursing Council of Kenya, Kenya Clinical Officers Council and Kenya Medical Practitioners and Dentist Board)
9. Representative of Private Hospitals (Kenya Healthcare Federation)
10. Representative of FBOs (Christian Health Association of Kenya (CHAK)
11. Chair, County Executive Committee Member for Health
12. Development Partners of Health in Kenya (DPHK); WHO, UNFPA, USAID, DFID, UNICEF, HENNET, JICA etc.
13. Inter-Religious Council of Kenya
14. Kenya National Human Rights Commission (KNHRC)

Leadership

The Director of Medical Services, chairs the committee and o-opts other members as need arises.

Terms of Reference for the National MPDSR Committee

Purpose of the National MPDSR Committee

The National Committee on Maternal, Perinatal Death Surveillance and Response (MPDSR) is a non-statutory, ministerial advisory committee established by the Cabinet Secretary of Health through a gazette notice.

The primary purpose of the National Committee is to provide oversight for all MPDSR activities in the country and to promote the notification, review and response to all maternal and perinatal deaths in Kenya. The committee will hold semi-annual meetings.

Roles and responsibilities of the National Committee

1. Provide oversight for all MPDSR activities.
2. Review/evaluate implementation of MPDSR across the various levels.
3. Review/approve the MPDSR reports and make recommendations.
4. Resource mobilization.

National MPDSR Secretariat

The National MPDSR Secretariat is based at the reproductive and maternal health services unit within the Division of Family Health. The composition of the MPDSR Secretariat include:

1. Head, Division of Family Health
2. Head, Reproductive and Maternal Health Services Unit
3. Maternal New-born Health, Monitoring and Evaluation program managers and new-born health
4. Head, New-born, Child and Adolescent Health Unit
5. Head, Health Management Information Systems
6. Disease surveillance and Response Unit representative

The secretariat will be coordinated by the head of the division of family health. Under the guidance of the National MPDSR committee, the functions of the MPDSR Secretariat include the following:

1. Coordination of MPDSR activities including its integration into existing systems and processes.
2. Review county surveillance reports and produce a six monthly summary report for key stakeholders.
3. Monitor progress of implementation of recommendations at national, county and facility level.
4. Promote the notification and response to all maternal deaths as well as the systematic review of perinatal deaths in Kenya.
5. Undertake regular evaluation of the MPDSR reporting system at national, county and facility level.
6. Produce a national MPDSR annual report.
7. Periodic update of MPDSR guidelines, tools and training materials.
8. Coordinate the collation and assessment of data on maternal and perinatal deaths.
9. Identify and train national maternal and perinatal death assessors who will review maternal and perinatal deaths through national review meetings annually.

10. Maintain a database of assessed maternal and perinatal deaths in the electronic Kenya Maternal Morbidity and Mortality Audit System (MaMMAS) software.
11. Build capacity of the MPDSR committee to produce regular national maternal death reports with key recommendations for national response.
12. Conduct or support operational research to generate evidence to improve implementation of MPDSR.

National Assessors

National MPDSR assessors are health care providers with speciality in obstetrics, paediatrics, midwifery, anaesthesia and public health. They are independent volunteers who are called upon to carry out confidential in-depth maternal and perinatal enquiries. The assessors will:

1. Conduct independent, detailed assessments of maternal deaths and correctly attribute cause of death based on WHO ICD-MM.
2. Explore and document the attributable/preventable factors to maternal and perinatal deaths.
3. Aggregate, analyse, interpret data and write reports on maternal and perinatal death.

The Response Cycle

MPDSR shall have feedback mechanisms at all levels. The National, County and Sub-County MPDSR reports shall be disseminated at all levels for information and record. Activities completed, as part of the response, to prevent avoidable deaths shall also be shared at all levels.

The purpose of feedback is to disseminate the key findings and recommendations of individual community or facility death reviews. It is also describing the plan of action to address avoidable factors contributing to the death(s). At the sub- country, county and national levels the feedback efforts are geared to inform the various stakeholders about reports from the higher levels. In providing feedback, death review findings, recommendation and the plan for the response should be disseminated to the facilities and communities where the data was collected, the feedback information should be in aggregated and de-identified form so that confidentiality is maintained for the people involved (both the deceased and their family members and the health providers). Finally, there should be safeguards to prevent the use of death review information from being used for litigation. Such safeguards may be in the form of strict adherence to confidentiality principles and de-identification of data in the reports. The safeguards may also include deliberate destruction of other documents that are review specific but have actual or potential identifiers after a designated time period – usually after the national CEMD team have completed the confidential enquiry.

Feedback or dissemination of reports can be in many formats depending on the intended audience. As a general principle, the more concise and brief the feedback document, the more likely it is to be read. In planning for feedback, it is critical that the content is tailored for the specific audience of stakeholders. Publication of entire national, county or sub-county report may be informative at the national, county and sub-county levels. However, for productive interaction with policy makers an executive summary of such reports is usually more appropriate and therefore recommended. At the lower levels, a poster

summarizing (national, county or sub-county) aggregated findings, recommendations and completed action plans disseminated from the higher levels to facility MPDSR committees will suffice. Generally, feedback to community MPDSR committees should be context specific and focusing on recent deaths in the community and what has been done to prevent similar future events. The local facility MPDSR committees collaborating with the reproductive health coordinators in the region will normally carry out the community feedback exercise.

CHAPTER 4: DATA MANAGEMENT AND M&E FRAMEWORK

MDSR systems stress delivery of real-time, systematic and frequent monitoring of maternal mortality levels, trends and causes of maternal death in communities and facilities. Monitoring and evaluation encompasses program monitoring and evaluation in the context of maternal neonatal and child health programs. To assess improved implementation of MPDSR in Kenya, a monitoring system is necessary to ensure that each step of the system is functional and improving with time. To assess the coverage and timeliness of information in the system that triggers response, indicators are tracked at both county and national level to determine whether the system is improving and resulting in a reduction of maternal and child deaths.

The aim of this chapter is to provide guidance on documentation and reporting of maternal and perinatal deaths at community, health facility and county level. This section also outlines how to overcome the challenges of incomplete, poor quality and untimely data and to outline how service providers and county health management teams can contribute towards enhanced evidence-based decision making.

M&E for MPDSR

Monitoring of the MPDSR system will be done using the indicators listed in Annex 1. Monitoring of the system is necessary to ensure the system is functioning adequately and improving with time. It is also important to have a monitoring system in place to assess timeliness and response so that decision makers can assess the status of the MPDSR system and make recommendations for improvement as needed. This will be done at both the county and national levels using data from the tools implemented at community and health facility level. A monitoring framework with indicators to be monitored nationally has been defined and is reviewed on an annual basis (Table 1).

Evaluation of the system is also done on a periodic basis (such as annually) when the MPDSR indicators demonstrate that one or more of the steps in the MPDSR process is not reaching expected targets, or if maternal mortality is not decreasing over time.

Data Quality for MPDSR

Having poor or unreliable data creates long-term costs and unforeseen effects compared with the costs and benefits of having good data. Practical and affordable strategies exist for generating timely and reliable data on health systems, but appropriate investment is needed to develop the capacity to collect, manage, analyse, disseminate and use information.

Information in the DHIS2 reporting system should ideally represent what happens at service delivery points to reflect high quality data. For this to happen the essential elements of completeness, timeliness, accuracy, reliability, precision, integrity and confidentiality must be true (REF: GoK 2015

Data Quality Review: Guidelines for Conducting Data Quality Reviews at All Levels). Data quality can be defined as the degree to which a data management system reflects the true situation of the information source.

As every data collection and management system is prone to errors resulting from data collection, processing and transmission it is of utmost importance to have data quality reviews. This process starts with a review of individual patient data at the community and health facilities for these errors to be minimized. Regular review of data is needed to validate the data being reported into the DHIS2 and to build trust in the data generated from the system. To do this health facilities and county health management teams should have monthly data review meetings in place to monitor maternal and perinatal deaths and provide recommendations to decision makers following the review. At the health facility level, the Facility in-Charge or a delegated member of the MPDSR committee will monitor the implementation of the remedial actions. Challenges and bottlenecks should be reported to the sub-county MPDSR committee to allow for redress. The monitoring results will be discussed during the following review meeting. At the county level, the RH Coordinator will monitor the implementation of the remedial actions received from the sub-counties. Challenges and bottlenecks should be reported to the national MPDSR committee to allow for redress. The monitoring results will be discussed during the following review meeting and feedback provided as necessary.

Data flow and use at various levels of the healthcare system

Mortality data consumption is necessary at all levels of the healthcare system for varying reasons with the end line being improvements in the health care system and eventual elimination of preventable maternal and perinatal deaths. Decision makers and stakeholders explicitly consider information in one or more steps in the process of policymaking, program planning and management or service provision.

Every level of health care not only needs to improve documentation and reporting to the next level, but also make data available and accessible in varied formats for routine use of data in decision making and performance review as summarized in Figure 1.

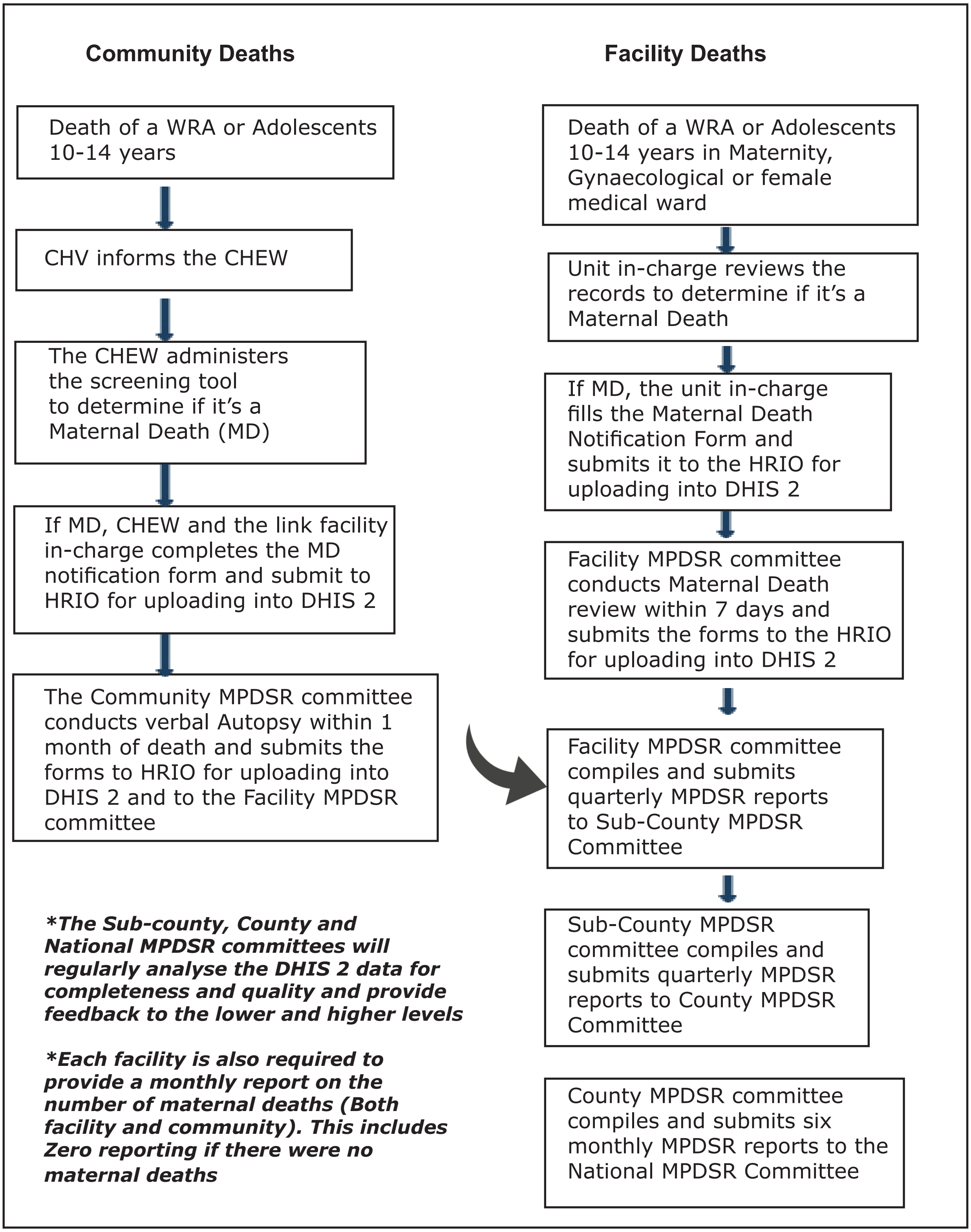

Figure 1. Flow chart for Maternal Death notification and review

Table 1: MPDSR reporting tools by service delivery level.

Data Tools	Who Fills	Duration	Copy sharing
Community Level			
Verbal autopsy (Maternal Death)	Trained Community Health Nurse or CHEW in consultation with the village health committee and the area Chief	Within 14 days of death occurrence	Filled in Quadruplicate and distributed as:<br>• Original to RMHSU and used for uploading into DHIS<br>• To CRHC<br>• To Facility MPDSR committee<br>• Remains with the Community Health Nurse/ CHEW in the community team
Community Perinatal Death report form	Nursing officer in-charge in consultation with immediate care givers	Within 72 hours of death occurrence	Filled in Quadruplicate and distributed as:<br>• Original to RMHSU and used for uploading into DHIS<br>• To CRHC<br>• To Facility MPDSR committee<br>• Remains with the Community Health Nurse/ CHEW in the community team
Facility Level: Hospitals, Health Centers, Dispensaries, Maternity Homes and Nursing Homes			
Maternal Death Notification Form (MDNF)	Unit in-charge in consultation with the immediate care giver(s)	Within 24 hours of death occurrence	Filled in Triplicate and distributed as:<br>• Original sent to the sub-county Public Health Nurse or RH coordinator<br>• To sub-county civil registrar<br>• Final copy remains in the Facility
Perinatal Death Review Form (PDR Form)	Nursing officer in-charge in consultation with immediate care giver(s)	Within 7 days of death occurrence	Filled in Quadruplicate and distributed as:<br>• Original to RMHSU and used for uploading into DHIS<br>• To CRHC<br>• To Facility MPDSR committee<br>• Final copy remains in the Facility

MPDSR Indicators

Level of monitoring	What to monitor (Indicator)	Indicators Definition	Data Source(s)	Indicative target (as per WHO guidelines)
**Overall system indicators**				
Sub-county	% of Sub counties with functional Facility MPDSR committees	**Numerator**: # of health facilities in a sub-county with functional review committees that hold meetings and record minutes<br><br>**Denominator**: Total # of facilities in a sub county	Sub- County MPDSR evaluation report	100%
County	% of Sub-Counties with functional Sub-county MPDSR committees	**Numerator**: # of Sub-counties with functional MPDSR committees that hold meetings and record minutes<br><br>**Denominator**: Total # of Sub-Counties within the County	County MPDSR evaluation report	100%
National	% of counties with functional MPDSR committees	**Numerator**: # of counties with functional MPDSR committees that hold meetings and record minutes<br><br>**Denominator**: Total # of Counties in Kenya	National MPDSR report	

Performance Indicators				
Community	% of community maternal deaths notified within 24 hours	**Numerator**: # of community maternal deaths notified within 24 hours<br><br>**Denominator**: # of community maternal deaths	DHIS2	>80%
Facility	% of maternal deaths notified within 24hrs	**Numerator**: # of maternal deaths in health facilities notified within 24 hours<br><br>**Denominator**: # of health facility maternal deaths	DHIS2	>90%
Facility	% of perinatal deaths notified within 72 hours	**Numerator**: # of perinatal deaths notified within 72 hours<br><br>**Denominator**: # of perinatal deaths at the health facility	DHIS2	>90%
Facility	% of Maternal deaths reviewed within 7 days and uploaded in DHIS	**Numerator**: # of facilities submitting completed MDR forms (timely, complete, accurate) that have been uploaded in 7 days<br><br>**Denominator**: # of maternal deaths in health facilities	DHIS2	100%
Facility	% Perinatal deaths reviewed within 7 days and uploaded in DHIS	**Numerator**: # of facilities submitting completed PDR forms (timely, complete, accurate) that have been uploaded in 7 days<br><br>**Denominator**: # of perinatal deaths in health facilities	DHIS2	100%

MNH indicators				
Facility/Sub-county/ County/National	Perinatal mortality rates at facility, sub-county, County and national	**Numerator**: Number of stillbirths and early neonatal deaths<br><br>**Denominator**: total number of births (stillbirths + live-births) in Facility/Sub-county/ County/National<br><br>***Note: PMR is calculated by dividing numerator by denominator and multiplying by 1000***	DHS/Vital statistics/ Service statistics	<15/1000
Facility/Sub-county/ County/National	Obstetric fatality rates of specific direct causes of maternal mortality	**Numerator**: # of deaths from obstetric compli¬cations in a health facility<br><br>**Denominator**: # of women suffering from obstetric compli-cations attended in the health facility	Health facility records	<5%
County	Number of maternal deaths by county	# of maternal deaths reported in communities and health facilities	DHIS2	
National	National Maternal Mortality Ratio	**Numerator**: # of maternal deaths<br><br>**Denominator**: Total number of live births Note: MMR is calculated by dividing numerator by denominator and multiplying by 100,000	DHS/Vital statistics	150/100000

**Response Indicators**				
Facility/County	Progress of action plans implementation at each level	Monitoring and Evaluation of progress of implementation of action plans developed	National MPDSR report	>80%
County	% of counties whose MPDSR reports are incorporated into the budgeting process	**Numerator**: # of counties whose MPDSR reports are incorporated into the budgeting process<br><br>**Denominator**: # of county MPDSR reports	County MPDSR evaluation report	>80%
National	National committee produces annual report	Annual MPDSR report produced and presented in parliament	National MPDSR report	100%

CHAPTER 5: LEGAL CONSIDERATIONS

Legal challenges encountered in establishing and implementing a MPDSR system represent a significant barrier to patients, families, health workers and facilities due to the lack of an enabling legal framework and minimal confidentiality and anonymity. The poor management of these issues results in fear of punitive measures and liability, poor transparency and quality of data. Very little information and research exists in this area, and further investigation and documentation of practical experiences is needed.

Identifying the challenges

Lack of legal framework

The presence of a legal framework is a driving force behind MPDSR systems and is critical to mitigating legal challenges on numerous levels including for the patient, family, health professional and facility (Pearson, 2009). An effective MPDSR system places emphasis on improving quality of care as opposed to placing blame on health staff.

Confidentiality and ethical considerations

Issues of confidentiality and privacy are a significant legal issue in terms of protection of women, families, health workers and review committees, including discussions and findings from the review process (Berg, 2011). The lack of autonomy, privacy, anonymity and immunity of patients, families, health professionals and review committees threatens the environment for a MPDSR system.

Autonomy: Women and families are should be fully informed of the purpose of the investigation and participation clearly outlined as voluntary.

Privacy: Privacy of the woman, her family and health workers is respected and maintained.

Anonymity: Details of the women, health care workers and facility are anonymised to prevent attribution of events to individuals.

Immunity: Protection for committee members, witnesses and others providing information should be provided for and concerns of liability and litigation addressed.

Access to information: Lack of access to and availability of sensitive and confidential medical materials (e.g. medical records, family and staff interviews) will have a significant impact on the understanding of events leading up to the death

Fear of liability and punitive measures

Many studies report concerns of liability, judgment, repercussions, blame and punitive measures for health professionals as a significant challenge to implementing and sustaining MDSR systems

(Hutchinson, 2010; Kongnyuy,2008; Konmgnyuy, 2009; Kalter, 2011; Gao, 2009). These concerns were reported as a result of a lack of legal framework to offer protection from punitive measures to health workers and committee members

A lack of a legal framework can lead to misconceptions and fears regarding possible punitive measures and the perception that audits are judgments on the actions of professional medical staff.

Poor transparency and quality of data

Underreporting and misreporting by health workers is likely to occur as a result of legal concerns including an overall lack of legal protection and fears of liability and punishment. During MDRs and PDRs, health professionals involved can react defensively, justifying actions and inactions, shifting the blame (e.g. blame death on the time taken to seek care) or even covering-up deaths or errors

Legal structures that support MPDSR

Review and understand the legal provisions and legal barriers in the MPDSR system, in order to facilitate implementation and expansion of the MPDSR.

Kenya has a supportive health policy framework, a National Reproductive Health policy and the National Quality Obstetrics and Perinatal Care guidelines, The MNH Road Map among other supporting policy documents that encourages the on-going investigation of all maternal deaths and facilitates the implementation process.

An MPDSR system should be incorporated as part of routine supervision and monitoring of maternal health outcomes as reflected in national policy.

Capacity building of MPDSR teams on the MPDSR guidelines, DHIS2 and a legal framework.

Establish structures on confidentiality

Ensure reporting mechanisms and forms are standardised and confidential (for the patient, family, health professional and facility), not just from 'outsider' access but also from reviewers (Gao, 2009) Women and families should be informed of the purpose of the investigation and that participation is voluntary.

The family and health workers must be assured that as much as possible, privacy will be maintained (WHO, 2004).

All individuals with access to any identifiable information should sign a confidentiality agreement, stating that they will not disclose any identifiable information.

Standardised tools such as Kenya Maternal Morbidity and Mortality Audit System (MaMMAS) software should be used for the MPDSR at the national level as well as a protocol for the facility level.

Data collection forms, case summaries, review meetings and any reports or dissemination of results should not contain personal identification.

Consent forms (or disclosure statements) should be administered prior to interviewing family members (World Bank, 2011).

Review all available information on each case and synthesise into case summaries for the committee, removing identifiers from records, and assign a ‘case number’ (CDC, 2001).

Aggregate findings from MPDSRs and remove identifiable information. Use findings to identify and respond to consistent trends.

Identifying information should be not be shared by electronic means

All committee members and staff should sign a confidentiality agreement before receiving any information on cases.

After committee meetings, all notes with identifying information collected for the purposes of the audit should be destroyed.

Ensure access to medical records and other sensitive and confidential information of events leading up to the deaths.

Establish a culture of non-blaming

The principle of ‘no name, no blame’ within MDSR, amongst health staff management and reviewers, should be established, supported and reiterated.

- Educate health professionals, that the MPDSR system seeks to identify improvements in the health care delivery system (at all levels) and use results for learning in order to improve quality of maternal care and not to provide the basis for litigating or punitive action.
- Establish immunity and legal protection for committee members, witness and others providing information from personal liability based on activities during the review process.
- Engage stakeholders within the planning and set-up process and educate on the ‘no-blame’ process and atmosphere.
- Hospital management should value and integrate the process, through providing leadership and human or financial resources.
- Surveillance and monitoring of implementation and processes to prevent misuse of MPDSR findings (e.g. confidentiality rules respected and punitive measures not introduced).
- Multi- professional approach taken to ensure every member of the team understands the MPDSR process, feels involved and valued.
- Communicate the change in accountability efforts and perspective from identification of individual responsible and blame to making a difference and contributing to improvements.
- Promote forums for sharing experiences of health workers and facilities, such as workshops and exchange visits.

IMPLEMENTATION PLAN

A concise implementation plan is essential for successful implementation of the Kenya MPDSR Guidelines 2016 and the activities that must be completed to facilitate the implementation. Critical among these is sensitization and training and related costs.

The key actor in implementation are the departments responsible for reproductive and maternal health both at the national (RMHSU) and county level. To successfully implement the MPDSR 2016 guidelines, the following will be needed:

- A costing process and allocation of funds for specific activities
- Development of a training manual for various stakeholders
- Training of Trainers who shall then escalate the trainings to the implementers.
- Training of various stakeholders at community, facility, sub-county, county and national levels.
- Mentorship of the various committees
- Monitoring and evaluation of the implementation of MPDSR

Other important aspects of implementation include: (a) Embedding the MPDSR principles in Kenyan Law (b) Development of accountability frameworks including performance contracts including MPDSR for managers of health services and (c) Development of an inter-operable program that includes an M&E dashboard to facilitate integration of data with existing systems such as DHIS, IDSR systems.

REFERENCES

1. World Health Organization, 2004. Beyond the Numbers: *Reviewing Maternal Deaths and Complications to make Pregnancy Safer.* ISBN 92 4 159183 8
2. World Health Organization, 2013. Maternal death surveillance and response Technical guidance information for action to prevent maternal death. ISBN 978 92 4 150608 3
3. WHO, International Statistical Classification of Diseases and Related Health Problems, Tenth Revision, Volumes 1 and 2 (Geneva: 1992).
4. N. Soleman, D. Chandramohan, and K. Shibuya, "Verbal Autopsy: Current Practices and Challenges," Bulletin of the World Health Organization 84 (2006): pp. 239–245.
5. Senanayake H, Goonewardene M, Ranatunga A, Hattotuwa R, Amarasekera S, Amarasinghe I. Achieving Millennium Development Goals 4 and 5 in Sri Lanka. BJOG 2011;118 (Suppl. 2):78–87.
6. WHO April 2014A Review of Progress in Implementation of the Commission on Information and Accountability for Women's and Children's Health: Kenya.
7. Federal Democratic Republic of Ethiopia: Maternal Death Surveillance and Response (MDSR), Technical Guideline. Addis Ababa Ethiopia. June 2013
8. Bacci, A. L. (2007). Introduction of Confidential Enquiries into Maternal Deaths and Near-Miss Case Reviews in the WHO European Region. *Reproductive Health Matters,* 15(30), 145-152.
9. Berg, C. (2011). From Identification and Review to Action – Maternal Mortality Review in the United States. *Seminars in Perinatology*, 36, 7-13.
10. Berg, C. D. (2001). Strategies to Reduce Pregnancy-Related Deaths: From Identification and Review to Actin. Atlanta: Centers for Disease Control and Prevention.
11. Danel, I. G. (2011). Maternal death surveillance and response. *Bulletin of the World Health Organisation*, 89, 779-779A.
12. Department of Health. (2012). *Saving Mothers 2008-2010: Fifth report on the confidential enquiries into maternal deaths in South Africa - Short report.* Republic of South Africa.
13. Gao, Y. K. (2009). Maternal mortality surveillance in an inland Chinese province. *International Journal of Gynaecology and Obstetrics,* 104, 128-131.
14. Graham, W. &. (2006). Universal reporting of maternal mortality: An achievable goal? *International Journal of Gynecology and Obstetrics*, 94, 234-242.
15. Group, S. A. (2008). Every death counts: use of mortality audit data for decision making to save the lives of mothers, babies and children in South Africa. *The Lancet*, 371, 1294-1304.
16. Hussein, J. &. (2012). Time for Actin: Audit, Accountability and Confidential Enquiries into Maternal Deaths in Nigeria. *African Journal of Reproductive Health,* 16(1), 9-14.
17. Hussein, J. (2007). Improve the use of confidential enquiries into maternal deaths in developing countries. *Bulletin of the World Health Organization,* 85, 68-69.
18. Hussein, J. D.-K.-T. (2009). Confidential inquiries into maternal deaths: Modifications and adaptations in Ghana and Indonesia. *International Journal of Gynecology and Obstetrics*, 106, 80-84.
19. Hutchinson, C. e. (2010). Exploring the sustainability of obstetric near- miss case reviews: a qualitative study in the South of Benin. *Midwifery*.

20. Kongnyuy, E. &. (2008). The difficulties of conducting maternal death reviews in Malawi. *BMC Pregnancy and Childbirth.*
21. Kongnyuy, E. M. (2009). Facility-based maternal death review in three districts in the central region of Malawi. *Women's Health Issues,* 19, 14-20.
22. Lewis, G. (2003). Beyond the Numbers: reviewing maternal deaths and complications to make pregnancy safer. *British Medical Bulletin*, 67, 27-37.
23. Lewis, G. (2008). Reviewing maternal deaths to make pregnancy safer. *Best Practice & Research Clinical Obstetrics and Gynaecology*, 22(3), 447-463.
24. Pathmanathan, I., et al. (2003). Investing in Maternal *Health: Learning from Malaysia and Sri Lanka.* Washington: The World Bank.
25. Pearson, L. d. (2009). Maternal death review in Africa. *International Journal of Gynaecology and Obstetrics,* 106, 89-94.
26. The World Bank. (2011). *Maternal Death Audit as a Tool Reducing Maternal Mortality*. The World Bank.
27. UNICEF. (2008). *Maternal and Perinatal Death Inquiry and Response.* UNICEF.
28. World Health Organization. (2004). *Beyond the Numbers: Reviewing maternal deaths and complications to make pregnancy safer.* Geneva: World Health Organization.

ANNEX 1: LIST OF CONTRIBUTORS

NAMES	DESIGNATION	ORGANIZATION
1. Dr. Wangui Muthigani	Program Manager, MNH	MOH- RMHSU
2. Prof. Edwin Were	Consultant	Moi University
3. Eunice Musembi	SDQA	MANI PROJECT
4. Wambua Willy	CRO	CRS
5. Dr. Njeri Nyamu	RH Specialist	MEVAL-PIMA
6. Dr. Jeanne Patrick	Program Manager -ASRH	RMHSU-MOH
7. Dennis Muya	Program Officer MNH	RMHSU-MOH
8. Dr. Chris Ouma	Program Officer	UNICEF NBI.
9. Martin Mating"I	Program Officer	NCAHU-MOH
10. Okoro Dan	Program Officer	UNFPA
11. Sylvia Gichuma	Technical Officer	LSTM (KENYA)
12. Peter Wainaina	HRIO	MOH
13. Ruth Muia	Program Officer	MOH
14. Dr. Were A.O	Technical Officer	LSTM
15. Dr. R.O Ogutu	OBSGYN/Resident	AKUHN
16. Ann. G. Mbabu	N.O	MOH-KIAMBU/ COUNTY
17. Annie Gituto	Program Officer	RMHSU
18. Joyce K. Onyango	Program Officer	RMHSU
19. Joan Okining	MNH-COORD.	SAVE THE CHILDREN
20. Kemboi A	N.O	MOH
21. Judith Maua	Senior Technical officer	LSTM
22. Magubo Mary	SNO	MOH-RMHSU
23. Zainab Lukhale	Lib/Asst.	MOH/RMHSU
24. Elizabeth Washika	Program Officer	MOH/RMHSU
25. Mercy Abuoro	SNO	MOH/KSM
26. Dr. Valeria Makory	SMO	MOH/Afya HSE
27. Rose Odeny	CRHC	MIGORI/MOH
28. Hambulle Mohammed	Program Officer	MOH/RMHSU
29. Joacquim Ogindo	Gynaecologist	NAKURU/PGH
30. Akaco Ekirapa	DDV Advisor	PIMA
31. Gathari Ndirangu	Technical Director	USAID/ MCSP
32. Joyce Lavussa	NPO/RH	WHO

33. Elizabeth Mgamb	Program Manager-M & E	MOH-RMHSU
34. Lynda Makayotto	M & E	DSRU
35. Benter Owino	RH Advisor	PIMA
36. Brian Kizito	RH specialist	PIMA
37. Samuel Cheburet	CRO	CRVSS
38. Pamela Godia	MPDSR Coordinator	LSTM
39. Dr. Ann Kihara	Chair, KOGS, Lecturer UON	KOGS, UON
40. Dr. Nelly Mugo	RH Lead Researcher	KEMRI
41. Agnes Gichogo	Technical Officer	LSTM
42. Prof Zahida Qureshi	OBGYN Professor	UON
43. Dr. Lynn Kanyuuru	RH Technical Advisor	USAID/MCSP

This Guideline has been produced
with technical and financial support
from UNFPA Kenya
