# Supplementary material for: Implementing Maternal Death Surveillance and Response in Kenya: Incremental Progress and Lessons Learned
Source: Glob Health Sci Pract. 2017 Sep 27;5(3):345–54. doi: 10.9745/GHSP-D-17-00130 (PMC5620333; doi:10.9745/GHSP-D-17-00130)
Supplement: Supplement 1 [file 17-00130-Smith-Supplement2.pdf]

NATIONAL MPDSR COMMITTEE

Core Members

1. Director of Medical Services
2. Head, Department of Preventive and Promotive Health Services
3. Head, Division of Family Health
4. Ministry of Health, Legal Advisor
5. Director Civil Registration Services
6. Representatives of Professional Organizations
    - Kenya Obstetrics and Gynaecological Society
    - Kenya Paediatric Association
    - Kenya Medical Association
7. Representatives of training institutions (one position)
    - College of Health Sciences, University of Nairobi, Chair OB/GYN Department
8. Regulatory Bodies
    - Nursing Council of Kenya
    - The Midwives Association, Kenyan Chapter
    - Kenya Clinical Officers Council
    - Kenya Medical Practitioners and Dentist Board
9. Representative of Private Hospitals.
    - Kenya Federation of Private Hospital
10. County Executive Committee Member for Health
11. Development Partners of Health in Kenya (DPHK); WHO, UNFPA, USAID, DFID,
12. Inter-Religious Council of Kenya
13. Kenya National Commission on Human Rights (KNCHR)

Terms of Reference for the National MPDSR Committee

Leadership

The Director of Medical Services, chairs the committee and co-opts other members as need arises.

Purpose of the National MPDSR Committee

The National Committee on Maternal, Perinatal Death Surveillance and Response (MPDSR) is a non-statutory, ministerial advisory committee established by the Cabinet Secretary of Health through a gazette notice.

The primary purpose of the National Committee is to coordinate all MPDSR activities at a central level and to promote the notification and response to all maternal and perinatal deaths in Kenya. The committee also oversees the writing of periodic Confidential Enquiry

into Maternal Deaths Reports that include practical recommendations for national response and submits the report to the Cabinet Secretary for Health.

Roles and responsibilities of the National MPDSR Committee:

- A Ministerial advisory committee established by the Cabinet Secretary of Health through a gazette notice.
- Provide oversight of all MPDSR activities in the country
- Promote the notification, review and response to all maternal and perinatal deaths in Kenya.
- Review/evaluate National MPDSR activities implementation
- Review/approve MPDSR reports and make recommendations
- Resource mobilization for maternal and newborn health
- Identifies and uses opportunities to embed MPDSR into the national Legal framework.
- Receives and adopts the Confidential Enquiry Report into Maternal Deaths from the MPDSR secretariat
- Submits a copy of the Confidential Enquiry Report into Maternal Deaths to the Cabinet Secretary, Ministry of Health for presentation to the Joint Senate and Parliamentary Committee on Health
- Advocates for continued visibility of maternal and perinatal health at the highest policy level as well as for resource mobilisation.
- Reviews and adopts TORs for the independent assessors
- Receives and appoints Independent Assessors from a list presented by the MPDSR Secretariat
- Meets semi annually

National MPDSR Secretariat

The National MPDSR Secretariat is based at the reproductive and maternal health services unit, Division of Family Health. **The composition of the MPDSR Secretariat include:**

1. Head, Division of Family Health
2. Head, Reproductive and Maternal Health Services Unit
3. Maternal Newborn Health and Monitoring and Evaluation program managers and officers
4. Head, Newborn, Child and Adolescent Health Unit
5. Head, Community Health Services Unit
6. Head, Health Management Information Systems
7. Head, Standard Quality and Regulations
8. Head, Health Promotion
9. Development partners and implementing partners
10. National MPDSR Coordinator
11. Administrative /Logistics Assistant

The secretariat will operate under the coordination of the National MPDSR Coordinator, a program officer and logistics officer. Under the guidance of the National MPDSR committee, the functions of the MPDSR Secretariat include the following:

1. Coordination of MPDSR including its integration with existing systems and processes
2. Review of county surveillance reports and produce six monthly summary reports
3. Monitor progress of implementation of recommendations at national, county and facility level.
4. Promote the notification and response to all maternal deaths as well as the systematic review of perinatal deaths in Kenya.
5. Undertake regular evaluation of the MPDSR reporting system at national, county and facility level
6. Produce a national MPDSR annual report
7. Periodic update of MPDSR guidelines, tools and training materials
8. Coordinate the collation and assessment of data on maternal and perinatal deaths;
9. To identify and train national maternal and perinatal death assessors who will review maternal and perinatal deaths through national review meetings annually.
10. Maintain a database of assessed maternal and perinatal deaths in the electronic Kenya maternal Morbidity and Mortality Audit System (MAMMAS) software
11. To build capacity of the MPDSR committee to produce regular National maternal death reports with key recommendations for national response
12. Conduce operational research to generate evidence to improve implementation of MPDSR
13. Advocate for the establishment of a legal framework that protects the entire MPDSR process as well as the assessors
14. To coordinate of all National MPDSR committee meetings, acting as secretariat to this committee

National Assessors

National MPDSR assessors are health care providers with speciality in obstetrics, paediatrics, midwifery, anaesthesia and public health. National assessors are selected to represent various bodies and institutions such as: KMDPB, KOGS, Nursing Council, the private health sector, teaching and referral hospitals and county hospitals. The assessors are trained to provide the following roles through regular national review meetings facilitated through the National MPDSR secretariat:

- to conduct independent, detailed assessments of maternal deaths and correctly attribute cause of death based on WHO ICD-MM
- use the assessors form to conduct detailed assessments of maternal deaths
- explore, document and find out the attributable/preventable factors to maternal and perinatal deaths
- aggregate, analyse, interpret data and write reports on maternal and perinatal deaths
